# Modulation of Cellular, Molecular, and Humoral Responses by PQ Grass 27,600 SU for the Treatment of Seasonal Allergic Rhinitis: A Randomised Double Blind Placebo Control Exploratory Field Study

**DOI:** 10.1111/all.16640

**Published:** 2025-07-08

**Authors:** Janice A. Layhadi, Sviatlana Starchenka, Pieter‐Jan De Kam, Elizabeth Palmer, Lily Y. D. Wu, Sean T. Keane, William T. Fulton, Prista Hikmawati, Xun Meng, Paulina Filipaviciute, Anna Cutrina Pons, Kemi Oluwayi, Katarzyna Lis, Oliver Armfield, Murray A. Skinner, Matthew D. Heath, Simon J. Hewings, Matthias F. Kramer, Mohamed H. Shamji

**Affiliations:** ^1^ National Heart and Lung Institute Imperial College London London UK; ^2^ Allergy Therapeutics PLC Worthing UK; ^3^ Bencard Allergie GmbH Munich Germany

**Keywords:** allergy treament, basic mechanisms, immune tolerance, immunotherapy and tolerance induction, immunotherapy clinical, immunotherapy vaccines and mechanisms, T cells

## Abstract

**Background:**

A short‐course pre‐seasonal subcutaneous injection of PQ Grass is clinically effective for the treatment of allergic rhinitis, though its mechanism remains unclear. The aim of the study was to interrogate immunological mechanisms induced by PQ Grass conventional and extended regimens.

**Methods:**

A RDBPC exploratory field study involving participants that either received injections of PQ Grass with a cumulative dose of 27,600 SU conventional (six once weekly injections) or extended regimen (three once weekly injections followed by three once monthly injections) or placebo containing microcrystalline tyrosine (MCT) (placebo + MCT) or saline (placebo) was performed. Humoral, cellular, and molecular responses were assessed at baseline (V1), end of treatment, prior to grass pollen season (V12) and end of pollen season (V15). Immunoglobulin analyses and cellular/gene microarray analyses were performed in the sub‐study cohort consisting of PQ Grass Conventional (*n* = 25 and *n* = 10, respectively), PQ Grass Extended (*n* = 26 and *n* = 10, respectively), Placebo with MCT (*n* = 13 and *n* = 5, respectively), and Placebo (saline; *n* = 12 and *n* = 5, respectively).

**Results:**

Both PQ Grass regimens, conventional and extended, were associated with improvement in total combined scores (TCS) with a relative difference of −35.0% (*p* = 0.03) and −40.8% (*p* = 0.01) against placebo with MCT, respectively. Both PQ Grass treatment regimens were associated with increases in the sIgG_4_/sIgE ratio (all, *p* < 0.05) and induction of IgA_1_ (all, *p* < 0.05) and IgA_2_ (all, *p* < 0.01) compared to placebo groups. Nasal fluid (*p* < 0.01) and serum (*p* < 0.05) blocking antibodies are functional and have the capacity to inhibit allergen‐IgE complex formation and binding to B cells in the PQ Grass groups. In vitro cellular and microarray gene analyses demonstrated that the extended PQ Grass regimen was more proficient in modulating the immune response towards a tolerogenic milieu by dampening pro‐inflammatory type 2 immune response and the associated cytokines (*p* < 0.05), immune deviation towards a Th1 response (*p* < 0.05), and induction of FOXP3^+^ Treg cells (*p* < 0.05).

**Conclusions:**

For the first time, we highlight differential mechanisms of tolerance induction by PQ Grass, with the extended regimen being superior in modulating T cell compartments.

**Trail Registration:**

Trial number: PQGrass309, EudraCT number: 2020‐000408‐13, Clinicaltrials.gov identifier: NCT04687059, and NCT05540717

## Graphical abstract

The aim of the study is to interrogate immunological mechanisms induced by PQ Grass conventional and extended regimens. A short‐course pre‐seasonal subcutaneous injection of PQ Grass is effective in inducing immune tolerance by generating functional blocking antibodies and modulating T cell compartments. PQ Grass involving the extended regimen is more superior to the conventional regimen in dampening Th2, while promoting Th1 and Treg responses. FOXP3, forkhead box protein P3; IFNG, interferon gamma; IL, interleukin; MCT, microcrystalline tyrosine; PQ Grass, Pollinex Quattro Grass; RDBPC, randomised double blind placebo control studies; SATB1, special AT‐rich sequence‐binding protein‐1; sIg, specific immunoglobulin; SU, standardised unit; Th, T helper cell; Treg, T regulatory cell; V, visit.

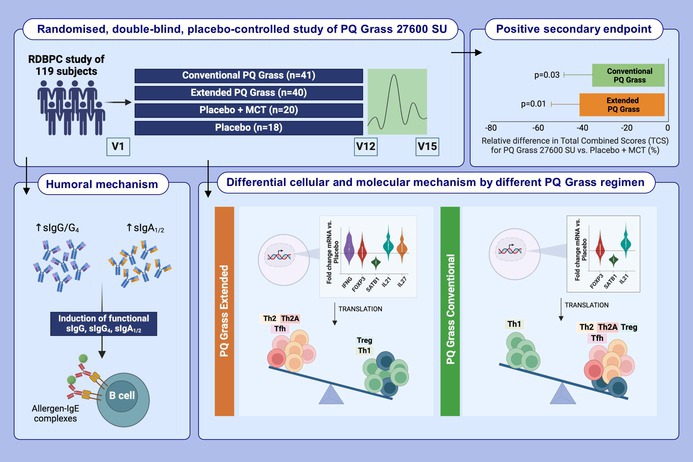

**Abbreviations**
AIT: allergen immunotherapy
AR: allergic rhinitis
BAT: Basophil activation test
Breg: B regulatory cells
CPT: conjunctival provocation test
CSMS: combined symptom and medication score
dMS: daily medication score
dSS: daily symptom score
ELISA: enzyme linked immunosorbent assay
GPS: grass pollen season
IL: interleukin
MC: metacluster
MCT: microcrystalline tyrosine
MPL: monophosphoryl Lipid‐A
PBMC: peripheral blood mononuclear cells
RDBPC: randomised double blind placebo control studies
SAR: seasonal allergic rhinitis
SCIT: subcutaneous immunotherapy
sIgA_1_
: specific immunoglobulin A_1_
sIgA_2_
: specific immunoglobulin A_2_
sIgG_4_
: specific immunoglobulin G_4_
SLIT: sublingual immunotherapy
SOM: self‐organising maps
SU: standardised unit
TCS: total combined score
Tfh: T follicular helper cells
Th2: T helper 2 cells
Th2A: allergen‐specific Th2 cells
Treg: T regulatory cells
TSS: total symptom score

## 1 Introduction

Allergic rhinitis (AR) with or without rhinoconjunctivitis is a chronic inflammatory condition of the nasal mucosa that can be triggered by inhalation of common seasonal or perennial allergens [1, 2]. AR is estimated to affect up to 40% of the global population with increasing prevalence in recent years [1, 3, 4]. It is associated with rhinorrhoea, sneezing, nasal itching, and congestion, all of which subsequently leads to significantly lower quality of life due to impaired sleep, learning difficulties, and work performance. These highlight their economic problem. The most common form of symptom reliever therapy is anti‐histamines and corticosteroid. In the subset of patients who do not respond to these therapies, allergen‐specific immunotherapy (AIT) is recommended. AIT, administered subcutaneously (SCIT) or sublingually (SLIT), remains to be the only disease‐modifying treatment that offers long‐term clinical benefit to allergic individuals [5].

Conventional AIT, when given for 3 years or more, has been associated with clinical benefit upon discontinuation of the treatment. This is accompanied by induction of blocking antibodies such as IgG, IgG_4_, IgA_1_, and IgA_2_. In a side‐by‐side study comparing grass pollen SCIT and SLIT, the Gauging Response in Allergic Rhinitis to Sublingual and Subcutaneous Immunotherapy (GRASS) trial revealed a differential induction of blocking antibodies following the two routes of administration. Whilst IgG and IgG_4_ were predominantly induced by SCIT, IgA_1_ and IgA_2_ were induced more by SLIT [6]. Furthermore, long‐term clinical efficacy of AIT has been associated with several mechanisms that include (1) immune deviation from a Th2 towards a Th1 response [7, 8, 9, 10], (2) dampening of Th2 and allergen‐specific Th2 (Th2A) cells [9, 11, 12, 13], (3) suppression of T follicular helper (Tfh) cells [14], and (4) induction of regulatory T (Treg) [12, 15, 16, 17, 18] and B (Breg) cells [14, 19, 20, 21]. Despite its efficacy, conventional AIT is far from optimal, with occasional severe side effects due to the use of whole unmodified allergen extract and poor patient compliance due to the need for a long treatment duration to achieve desensitisation and tolerance. These challenges could be improved using a modified allergen extract with disrupted IgE epitopes, making them hypoallergenic and thus safer for use in SCIT, and also administration of modified short‐course SCIT involving only six pre‐seasonal injections, which will improve patient compliance.

PQ Grass contains an extract of 13 grass pollen species from *Pooideae* spp. and has been developed as a pre‐seasonal SCIT product. The allergen extract is chemically modified by means of glutaraldehyde cross‐linking, generating a polymeric allergoid. This modification preserves immunogenic properties whilst reducing allergenicity [22, 23, 24]. Furthermore, favourable immunogenic characteristics of the allergoid further enhanced by an adjuvant system consisting of microcrystalline tyrosine (MCT) and monophosphoryl lipid‐A (MPL), where MCT acts as a depot adjuvant to prolong immune exposure whilst enhancing specific B cell responses and MPL serves as a toll‐like receptor 4 activator, facilitating Th1 immune responses [25, 26, 27].

PQ Grass showed favourable safety and efficacy profiles in the earlier‐phase clinical trials [28, 29, 30, 31]. A previous Phase II dose‐finding study of PQ Grass demonstrated a significant dose–response relationship with the primary total symptom score (TSS), measured during a post‐treatment conjunctival provocation test (CPT) [32]. The study established a cumulative dose of 27,600 standard units of PQ Grass (also referred to as PQ Grass 27,600 SU) delivered by 6 injections as the optimally effective and safe dose to carry into a Phase III. Although a strong efficacy dose response was observed in Phase II, the underlying mechanism of immunotherapy with PQ Grass remains to be fully elucidated. Unravelling its mechanism of action would allow the identification of biomarkers specific for PQ Grass, which could then be further validated in other clinical studies to assess their suitability in predicting individual AIT response and efficacy. Most importantly, a biomarker footprint specific for PQ Grass may support a personalised medicine approach, providing a way to predict treatment response for individual patients.

In this study, we investigated the mechanism of action of AIT using PQ Grass through a series of cellular, molecular, and humoral biomarker analyses on a sub‐study cohort collected from an exploratory RDBPC field study that form part of a staged Phase III clinical development program using a short‐course six pre‐seasonal injections of PQ Grass with a cumulative dose of 27,600 SU (conventional or extended regimen). De Kam et al. [33] reported earlier that both regimen approaches were effective and an improvement of up to 40% in the primary symptom and medication score (CSMS) were observed in the PQ Grass arms compared to placebo following only six pre‐seasonal PQ Grass injections. Primary efficacy endpoint results were further supported by improvement in secondary endpoint total combined score (TCS) for PQ Grass treatment groups compared to both placebo groups, which were demonstrated in this study. Our biomarker sub‐study revealed that both PQ Grass SCIT regimens induced local and systemic blocking antibodies that are functional and can inhibit the formation of allergen‐IgE complexes and their subsequent binding to B cells. Moreover, six pre‐seasonal PQ Grass injections are sufficient in modulating the immune response towards a tolerogenic milieu by dampening pro‐inflammatory Th2A response, immune deviation towards a Th1 response, and by inducing FOXP3^+^ Treg cells. Beneficial modulation of the immune response by PQ Grass was further emphasised by observations from microarray gene expression analyses which demonstrated dampening of pro‐allergic responses induced by type 2 cytokines and induction of regulatory profile indicated by Treg‐associated genes. In particular, PQ Grass elicits a novel regulatory subset of T_FH_ cells with the capacity to dampen the induction of B cell class‐switching to IgE. Overall, findings from these biomarker analyses outline cellular, molecular, and humoral changes that supports the efficacy of PQ Grass 27,600 SU for the effective treatment of SAR.

## 2 Methods

### 2.1 Subjects

**FIGURE 1:**
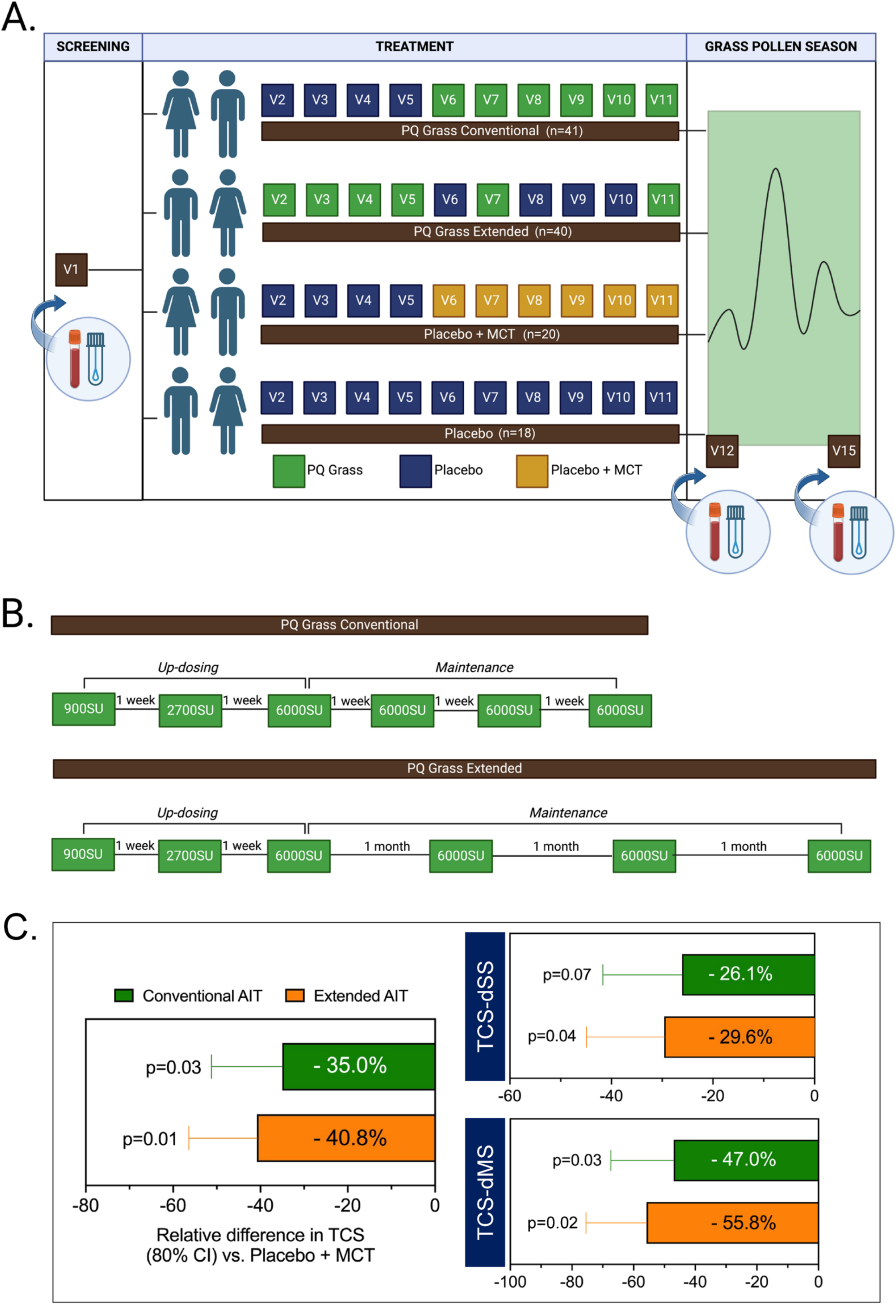
PQ Grass administered either as a conventional or extended regimen demonstrated a positive and efficacious clinical endpoint. (A) Schematic representation of the clinical trial design, indicating the four different treatment arms, the treatment received at each visit (arrows), and the corresponding visits in relation to the grass‐pollen season (GPS). (B) Dosing regimen for conventional and extended AIT treatment regimens. (C) Relative difference in Total Combined Scores (TCS), TCS‐dSS and TCS‐dMS of PQ Grass conventional or extended AIT groups compared to placebo with MCT. Sample size for each group is as follow: *n* = 41, 40, 20 and 18 for PQ Grass conventional, PQ Grass extended, placebo with MCT and placebo regimens, respectively. dMS, daily medication score; dSS, daily symptom score.

A total of 119 subjects were recruited into this randomised, parallel‐group, double‐blind, placebo‐controlled (RDBPC) exploratory field study conducted at 14 sites across the US and Germany. Subjects were randomised to one of four treatment arms in a 2:2:1:1 ratio (PQ Grass conventional regimen, PQ Grass extended regimen, placebo with MCT or placebo (saline), respectively) (Figure 1A). Subjects for the biomarker sub‐study were selected randomly from the pool of eligible subjects at the ratio 2:2:1:1. PQ Grass groups received active injections of 900, 2700, 6000, 6000, 6000, and 6000 SU administered sequentially. For the PQ Grass conventional regimen, three up‐dosing and three maintenance injections were administered once weekly, whilst for the PQ Grass extended regimen, three up‐dosing doses were administered once weekly, and three maintenance injections were administered once monthly (Figure 1B). Immunoglobulin analyses were performed in the sub‐study cohort consisting of PQ Grass Conventional (*n* = 25), PQ Grass Extended (*n* = 26), Placebo with MCT (*n* = 13) and Placebo (saline; *n* = 12). Cellular analyses and gene microarray analyses were performed on a subset of the sub‐study cohort consisting of PQ Grass conventional (*n* = 10), PQ Grass extended (*n* = 10), placebo with MCT (*n* = 5) or placebo (saline) (*n* = 5).

All subjects signed an informed consent form (ICF). Subjects, who participated in the biomarker sub‐study, signed an additional optional biomarker ICF which includes gene expression analyses studies. The clinical study was designed and conducted in accordance with the Declaration of Helsinki and in compliance with good clinical practice guidelines. Applicable US and EU regulatory authorities and ethical committees approved the study protocol and amendments. The study complied with the EU General Data Protection Regulation and the US Health Insurance Portability and Accountability Act.

### 2.2 Isolation of Human Peripheral Blood Mononuclear Cells (PBMCs)

Peripheral blood mononuclear cells (PBMCs) were isolated from fresh 60 mL heparinised venous blood by density gradient centrifugation technique. Heparinised blood was diluted 1:1 with RPMI‐1640 media (Invitrogen, Paisley, UK) and layered on 30% Ficoll‐Paque Plus (GE‐Healthcare, UK) and centrifuged for 25 min at 2200 rpm. The PBMC layer was collected, washed, and resuspended in RPMI‐1640. Viable cells were counted on a haemocytometer, using the trypan blue exclusion method.

### 2.3 In Vitro Humoral, Cellular and Molecular Biomarker Assays

Details outlining methodologies involved in ELISA, in vitro B cell, T cell and Treg stimulation assays can be found in the online repository method section. Methods outlining gene expression microarray analysis can also be found in the online repository method section.

### 2.4 Statistical Analysis

Results were analysed using GraphPad Prism 9 software (GraphPad Software, USA). Correlation analyses were performed using Spearman's rank test. Statistical evaluation between‐group and within‐group was performed using the two‐tailed Mann–Whitney test and Wilcoxon matched‐pairs signed rank test, respectively. As this work entails exploratory analysis, no adjustment for multiple testing was performed. P values less than 0.05 were statistically significant.

## 3 Results

### 3.1 Demographics and Baseline Characteristics

Overall, 114 (95.8%) subjects completed the study. Five subjects prematurely discontinued the study with four subjects (9.8%) from the PQ Grass conventional group and one subject (2.5%) from the PQ Grass extended group. (Figure S1). Four treatment groups were comparable with respect to the most demographic variables. All subjects had allergic rhinitis (100%) and/or allergic conjunctivitis (80.7%). At screening baseline, all subjects had grass‐specific IgE by ImmunoCAP class ≥ 2 (Table S1). Baseline demographics for biomarker sub‐study subjects across all four treatment groups were also comparable with 29 subjects (96.7%) reported allergic rhinitis and 19 (63.3%) allergic conjunctivitis at the start of the study. All biomarker subjects had positive SPT to 12 grass mix (≥ 3 mm) and grass‐specific IgE by ImmunoCAP class ≥ 2 (Table S2).

### 3.2 PQ Grass, Administered Either as a Conventional or Extended Regimen, Provides an Efficacious Treatment With the Positive Secondary Endpoint

The efficacy of 27,600 SU of PQ Grass administered either as a conventional or extended regimen was assessed using TCS during the peak grass pollen season (GPS). In the whole study cohort, improvement in TCS during the peak GPS was observed in both PQ Grass conventional and extended regimens with a relative difference of −35.0% (80% CI: −51.2% to −18.9%) (*p* = 0.03) and −40.8% (80% CI: −56.4% to −25.1%) (*p* = 0.01) against placebo with MCT, respectively (Figure 1C, Table S3). A similar observation was seen in relative difference for daily symptom score (dSS) and daily medication score (dMS). Improvement in TCS over the peak GPS was also demonstrated for both PQ Grass treatment regimens when compared to placebo group (Figure S2). In all the secondary endpoint readouts, a superior efficacy was seen in the PQ Grass extended regimen compared to conventional regimen (Figure 1C, Figure S2). In the sub‐study biomarker cohort, a similar trend was observed whereby an improvement in TCS during the peak GPS was observed in both PQ Grass conventional (*n* = 10) and extended (*n* = 10) groups with a relative difference of −29.7% (80% CI: −64.1% to 4.7%) and −46.9% (80% CI: −77.1% to −16.7%) against placebo with MCT (*n* = 5), respectively (Table S4). Reduction in TCS for sub‐study biomarker cohort in PQ Grass conventional (−31.9% [80% CI: −65.1% to 1.3%]) and PQ Grass extended groups (−48.6% [80% CI: −77.4% to −19.7%]) was also demonstrated vs. placebo group (Table S4).

### 3.3 Both Conventional and Extended Regimen of PQ Grass Are Associated With the Induction of Blocking Antibodies Locally and Systemically

**FIGURE 2:**
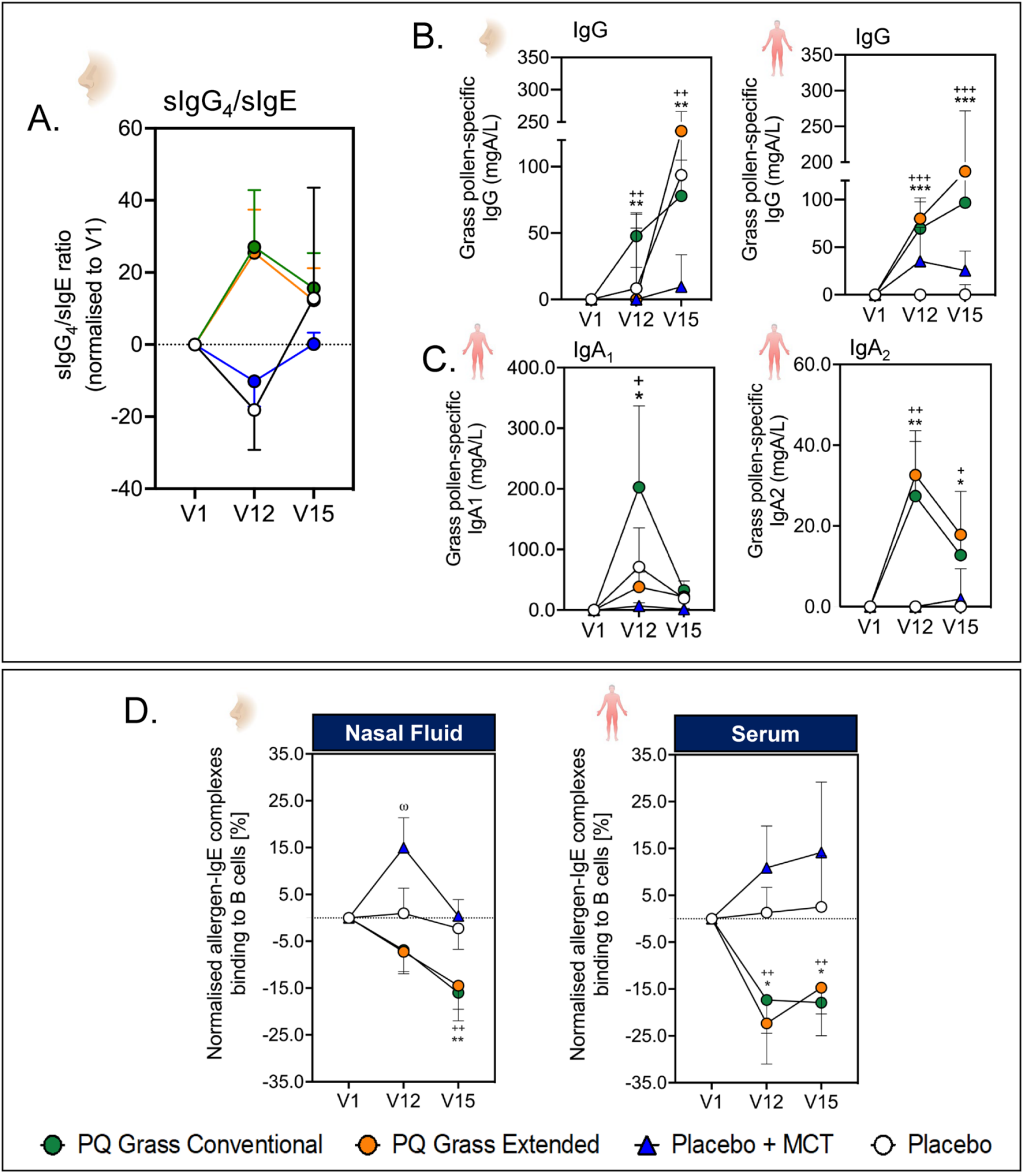
Conventional and the extended PQ Grass regimens have the capacity to induce functional blocking antibodies in the local target organ and systemically. (A) The ratio of grass pollen‐specific IgG_4_/ grass pollen‐specific IgE for conventional and extended AIT groups and the placebo groups for visits 1, 12 and 15 was measured in extracted nasal fluid. (B) Grass pollen‐specific IgG measurement in the PQ Grass conventional and extended regimens and the placebo groups for visits 1, 12 and 15 in nasal fluid (left) and serum (right). (C) Grass pollen‐specific IgA_1_ (left) and grass pollen‐specific IgA_2_ (right) measurement of the PQ Grass conventional and extended regimens and the placebo groups for visits 1, 12 and 15 in serum. (D) Percentage of grass pollen allergen‐IgE complexes bound to CD23 on B cells for the conventional and extended AIT groups and the placebo groups for visits 1, 12 and 15 in nasal fluid (left) and serum (right). Immunoglobulin analyses were performed in the sub‐study cohort consisting of PQ Grass Conventional (*n* = 25), PQ Grass Extended (*n* = 26), Placebo with MCT (*n* = 13) and Placebo (saline; *n* = 12). Data are presented as mean ± SEM. *p < 0.05, **p < 0.01 and ***p < 0.001, Mann–Whitney *U* test for between group comparison. *, + and ω denotes significance V12 and V15 compared to V1 in conventional AIT, extended AIT, and placebo + MCT, respectively.

A successful AIT has been associated with the induction of functional blocking antibodies that can compete with IgE, subsequently inhibiting activation of the downstream pro‐inflammatory allergic responses. The effect of PQ Grass on levels of antibodies in the serum and nasal fluid was assessed by quantifying grass pollen‐specific IgG (sIgG), IgG_4_, IgA_1_, IgA_2_ and IgE at V1, V12 and V15. A trend of time‐dependent induction in the ratio of nasal sIgG_4_/sIgE was observed in PQ Grass conventional and extended regimen groups at V12 and V15 and not in the placebo groups (Figure 2A). Moreover, a time‐dependent induction in the level of grass pollen‐specific sIgG (all, *p* < 0.001; Figure 2B) was observed in PQ Grass conventional compared to V1, in both serum and nasal fluid. Those who received PQ Grass extended demonstrated induction of grass pollen‐specific IgG at V12 and V15 in serum and nasal fluid, respectively (all, *p* < 0.01; Figure 2B). Groups that received placebo with MCT demonstrated a subtle increase in serum sIgG induction, whilst those that received placebo alone demonstrated induction of nasal sIgG at V15 (Figure 2B) though none of these observations were statistically significant. Significant differences were also observed between study treatment groups in the level of grass pollen serum sIgG, which was found significantly higher in the PQ Grass conventional compared to placebo with or without MCT at V12 (*p* = 0.0119 and *p* = 0.0010, respectively) and at V15 (*p* = 0.0262 and *p* = 0.0052, respectively) (Table S5). Within the PQ Grass extended AIT, grass pollen serum sIgG was significantly higher at V12 compared to placebo with or without MCT (*p* = 0.0059 and *p* = 0.0004, respectively) and at V15 (*p* = 0.0037 and *p* = 0.0003, respectively).

In addition to the induction of sIgG, a transient induction of serum grass‐specific sIgA_1_ was observed at V12 in the conventional AIT group (*p* = 0.0124; Figure 2C) compared to V1. A very subtle, yet significant, induction in the level of sIgA_1_ was also observed at V12 in the extended AIT group (*p* = 0.0484; Figure 2C) compared to V1. Similarly, induction in the level of grass pollen sIgA_2_ in serum was observed in both the conventional and extended AIT groups at V12 (both, *p* < 0.01) and V15 (both, *p* < 0.05), compared to V1 (Figure 2C, right panel). No significant induction was observed at V12 and V15 in the placebo groups compared to V1. Significant differences were also observed between study cohorts (Tables S6 and S7). A significant induction of serum grass‐specific sIgA_1_ was seen in conventional AIT and extended AIT treatment groups compared to placebo with MCT at V12 (*p* = 0.0118 and *p* = 0.0408, respectively) and V15 (*p* = 0.0288 and *p* = 0.0139, respectively). Similarly, a significant induction of serum grass‐specific sIgA_2_ was seen in PQ Grass conventional regimen compared to placebo with or without MCT at V12 (*p* = 0.0013 and *p* = 0.0017, respectively) and in PQ Grass extended regimen compared to placebo with or without MCT (both, *p* = 0.0031). This induction was maintained in both PQ Grass conventional and extended groups compared to placebo at V15 (*p* = 0.0381 and *p* = 0.0270, respectively). Interestingly, the induction in the level of grass pollen specific nasal IgG, IgA_1_ and IgA_2_ did not reach statistical significance between any of the treatment groups in the local compartment.

### 3.4 Blocking Antibodies Induced Following PQ Grass Conventional and Extended Treatment Regimens Share a Similar Inhibitory Capacity

To assess the functionality of blocking antibodies induced following AIT treatment with PQ Grass, the IgE‐FAB assay, an in vitro surrogate model of facilitated allergen presentation to B cells [34], was performed. Antibodies generated within the local target organ (nasal fluid) and systemically (serum) displayed strong inhibitory capacity in preventing the cooperative allergen‐IgE complexes from binding to B cells. In the nasal mucosa, this inhibition was observed at both V12 and V15 compared to V1, though significance was only reached at V15 for both PQ Grass groups (PQ Grass conventional, *p* = 0.0179 and PQ Grass extended, *p* = 0.0105; Figure 2D, left panel). The inhibitory capacity of antibodies found within the serum was much more potent at V12 and was maintained up to V15 in both PQ Grass conventional (*p* = 0.0367 and *p* = 0.0081, respectively) and PQ Grass extended (*p* = 0.0491 and *p* = 0.0067, respectively) (Figure 2D, right panel), compared to V1. No inhibition of allergen‐IgE complexes binding to B cells was observed at any of the time points in both placebo groups compared to V1. Between‐group comparison also revealed a significant difference in the inhibitory capacity of nasal fluid and serum from conventional and extended AIT groups compared to placebo with MCT (Table S8).

### 3.5 PQ Grass Can Modulate Cellular Responses by Suppressing Pro‐Inflammatory T Cell Responses

**FIGURE 3:**
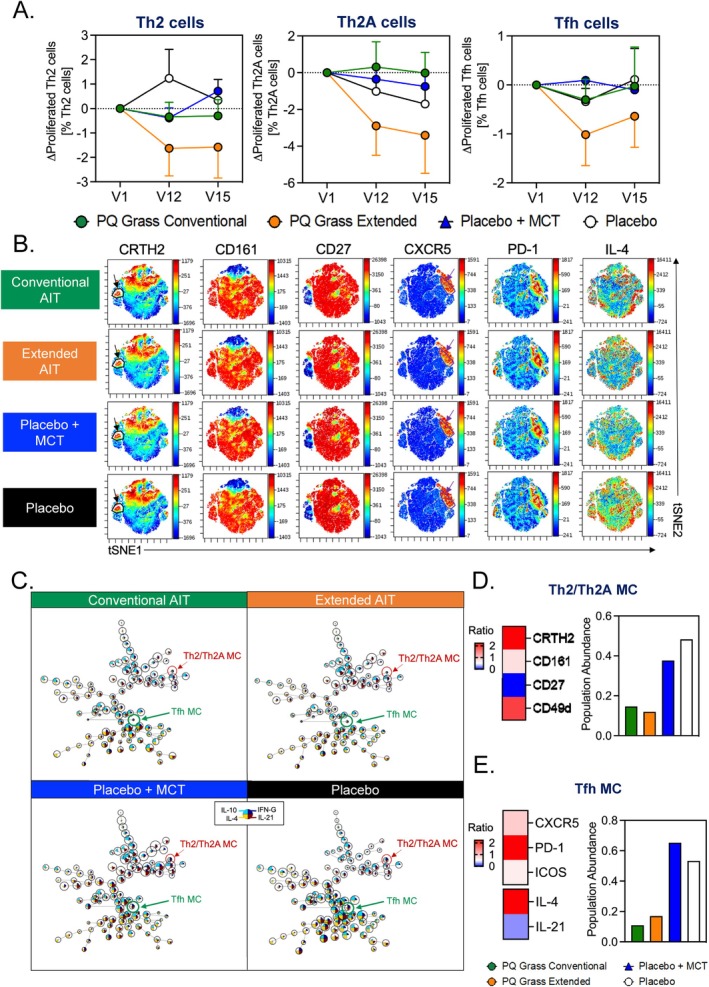
The extended PQ Grass regimen demonstrates suppression of pro‐inflammatory T cell responses. (A) Graphical representations of the percentage of proliferating Th2, Th2A and Tfh cells for the PQ Grass conventional and Extended groups and the placebo groups for visits 1, 12 and 15, normalised to visit 1. (B) Unbiased clustering analysis using viSNE was performed on flow cytometry data to compare expression of Th2/Th2A (black arrow) and Tfh cells (purple arrow) for both conventional and extended AIT and the placebo groups. (C) FlowSOM analysis highlighting the Th2/Th2A and Tfh metaclusters for each of the conventional and extended AIT groups and the placebo groups. (D and E) Population abundance of (D) Th2/Th2A metacluster and (E) Tfh metacluster for each PQ Grass conventional and extended AIT groups and placebo groups, with Th2/Th2A and Tfh metacluster phenotypes represented as a heatmap. Cellular analyses were performed on a subset of the sub‐study cohort consisting of PQ Grass conventional (*n* = 10), PQ Grass extended (*n* = 10), placebo with MCT (*n* = 5) or placebo (saline) (*n* = 5). Data are presented as mean ± SEM.

T cells are crucial in driving allergic inflammation, with Th2, Th2A and Tfh cells having been demonstrated to be the key players. To assess the mechanism of tolerance induction by PQ Grass, their effect on T cell responses in vitro was investigated in a subset of the biomarker sub‐study cohort. At V1, proliferation of Th2, Th2A and Tfh cells was observed in a dose‐dependent manner upon grass pollen allergen stimulation (Figure S3, Table S9). No significant changes in the level of proliferation of Th2, Th2A and Tfh cells were observed at V12 and V15 in PQ Grass conventional group and both placebo groups (Figure 3A). However, a trend of reduced proliferation of Th2, Th2A and Tfh cells can be seen at V12 and V15, compared to V1 in the PQ Grass extended group. These observations were confirmed through unbiased clustering analyses, viSNE and FlowSOM. The clustering algorithm using viSNE reveals a single island corresponding to Th2/Th2A cells and a separate island corresponding Tfh cells (Figure 3B). Further analysis using FlowSOM confirmed the presence of a single metacluster (MC) of cells with Th2/Th2A‐like phenotype (CRTH2^+^CD161^+^CD49d^+^CD27^−^) and another MC with Tfh‐like phenotype (IL‐4^+^CXCR5^+^PD‐1^+^ICOS^+^) (Figure 3C–E). Quantification of population within each of these MC demonstrated reduced abundance in those that received PQ Grass conventional and extended treatment regimen, albeit differential magnitude, compared to placebo groups (Figure 3D,E). These analyses therefore provide early evidence of the potential differential mechanism mediated by conventional or extended AIT regimen on pro‐inflammatory T cell compartment.

### 3.6 Immune Deviation Towards Tolerogenic T Cells Were Observed Following Extended Regimen and Conventional Regimen of PQ Grass, Respectively

**FIGURE 4:**
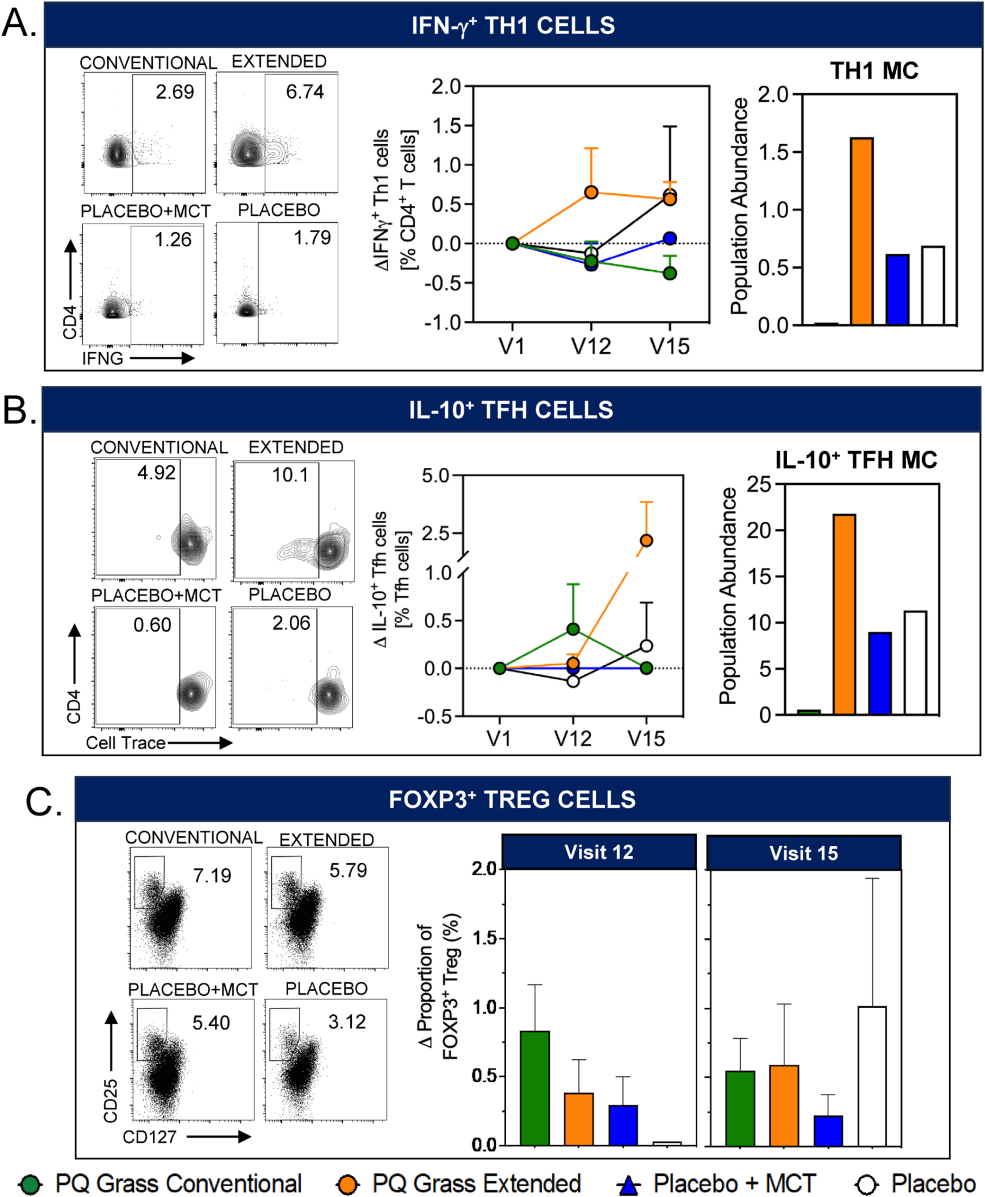
The conventional and the extended PQ Grass regimens promote immune deviation towards tolerogenic T cells. (A and B) Flow cytometry representative plots (left) of (A) IFN‐𝛄^+^ Th1 cells and (B) IL‐10^+^ Tfh cells, for both PQ Grass conventional and extended AIT groups and the placebo groups, with corresponding graphical representations (middle) of the percentage (A) IFN‐𝛄^+^ Th1 cells and (B) IL‐10^+^ Tfh cells for each treatment group at visits 1, 12 and 15. FlowSOM population abundance analysis at visit 15 (right) for metaclusters representing (A) IFN‐𝛄^+^ Th1 cells and (B) IL‐10^+^ Tfh cells for each treatment group. (C) Flow cytometry representative plots (left) of CD4^+^CD25^+^CD127^−^FOXP3^+^ Tregs and bar chart representation (right) of the percentage of FOXP3^+^ Tregs at visit 12 for both conventional and extended AIT groups and placebo groups. Cellular analyses were performed on a subset of the sub‐study cohort consisting of PQ Grass conventional (*n* = 10), PQ Grass extended (*n* = 10), placebo with MCT (*n* = 5) or placebo (saline) (*n* = 5). Data are presented as mean ± SEM.

A dampening of pro‐inflammatory Th2 response following an efficacious AIT treatment is often accompanied by immune deviation towards a tolerogenic response. To further elucidate the cellular mechanism of tolerance induction by PQ Grass AIT, the frequency of Th1, IL‐10^+^ Tfh and Treg cells was enumerated over the course of treatment in a subset of the biomarker sub‐study cohort. Extended AIT regimen, but not conventional AIT regimen, resulted in a trend towards heightened frequency of IFN‐y^+^ Th1 cells at V12, which persisted until V15 (Figure 4A, left and middle panel). Both placebo groups induced little to no change in the proportion of IFN‐y^+^ Th1 cells. This observation was also confirmed using unbiased clustering analysis, FlowSOM, whereby population abundance of MC corresponding to Th1 cells was elevated predominantly in those receiving extended AIT at V15 (Figure 4A, right panel). Interestingly, both PQ Grass conventional and extended AIT treatment regimens have the capacity to induce IL‐10^+^ Tfh cells, albeit not significant and displaying a different kinetic in response (Figure 4B). Whilst conventional AIT subtly elevated IL‐10^+^ Tfh cells at V12, which did not persist at V15, extended AIT induced a much more prominent response of IL‐10^+^ Tfh cells at V15 (Figure 4B left and middle panel). This was confirmed using FlowSOM analysis at V15 (Figure 4B, right panel). Finally, FOXP3^+^ Treg cells were induced predominantly by conventional AIT, but not extended AIT, at V12 (Figure 4C). These observations highlight that whilst both modes of the PQ Grass AIT regimen can modulate the T cell response towards immune deviation and tolerance, they exhibit very different profiles of cellular response and kinetics.

### 3.7 Microarray Analyses Reveal Distinct Repertoire of Gene Signatures Targeted by Conventional and Extended Regimen of PQ Grass

**FIGURE 5:**
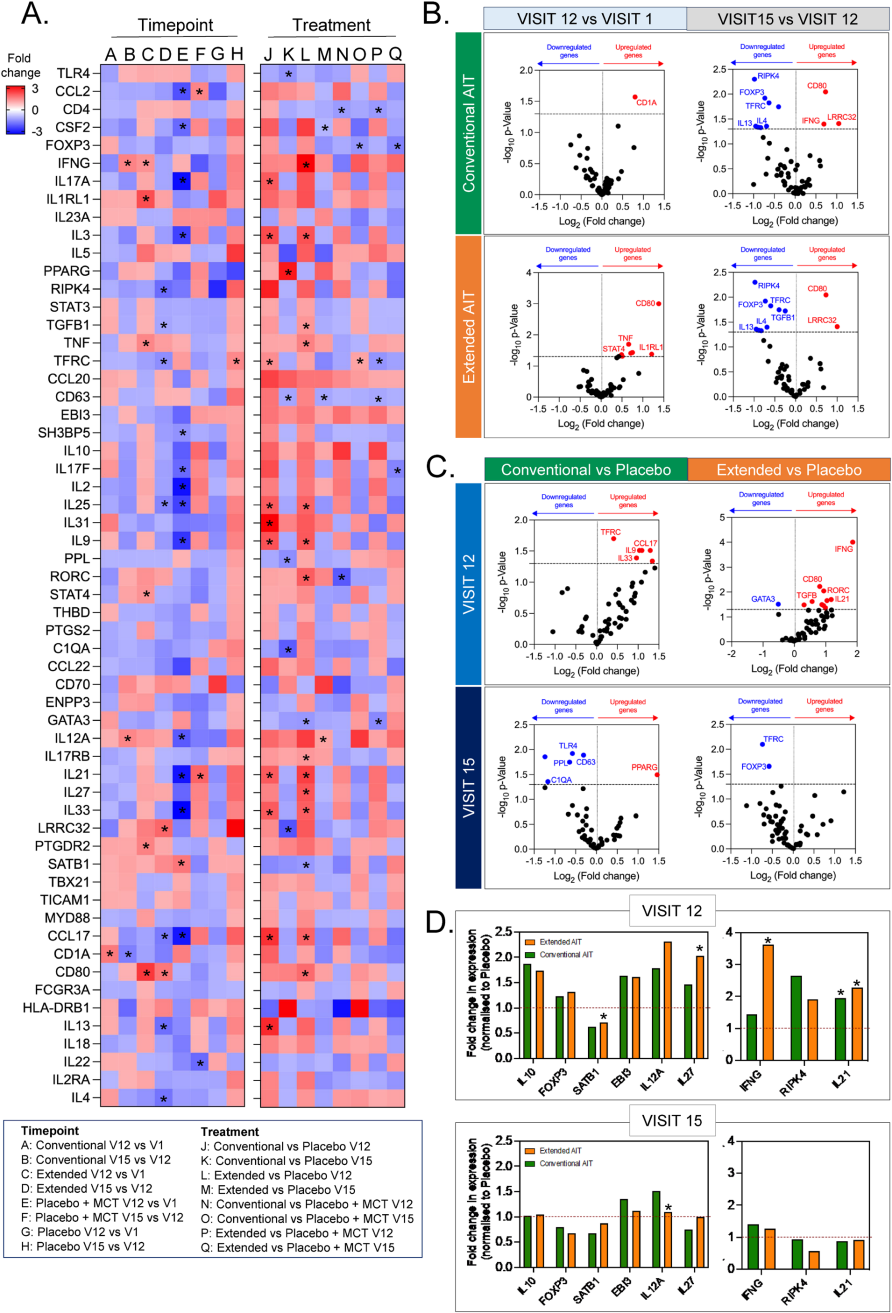
Distinct gene signatures targeted by conventional and extended PQ Grass regimens. (A) Heatmap of the microarray analysis depicting the fold change of the predetermined immune and inflammation‐related gene set between different timepoints within the same treatment group, labelled A‐H, and between treatment groups at the same timepoint, labelled J‐Q. (B) Volcano plot displaying the significantly upregulated and downregulated genes at visit 12 compared to visit 1 (left) and visit 15 compared to visit 12 (right) in the PQ Grass Conventional (top) and PQ Grass Extended (bottom) groups. (C) Volcano plot displaying the significantly upregulated and downregulated genes in the PQ Grass Conventional group compared to placebo (left) and PQ Grass Extended group compared to placebo (right) at visit 12 (top) and visit 15 (bottom). (D) Fold change of expression of regulatory T cell‐associated genes (left) and other immunomodulatory‐associated genes (right) for both PQ Grass Conventional and Extended groups compared to placebo without MCT at visit 12 (top) and visit 15 (bottom). Gene microarray analyses were performed on a subset of the sub‐study cohort consisting of PQ Grass conventional (*n* = 10), PQ Grass extended (*n* = 10), placebo with MCT (*n* = 5) or placebo (saline) (*n* = 5). Data are presented as mean ± SEM. *p < 0.05, Mann–Whitney *U* test for between‐group comparison.

Microarray analyses to investigate the effect of PQ Grass at the molecular level were performed on a set of pre‐determined genes associated with immune response and inflammation. The time course of treatment and treatment regimen resulted in the modulation of various genes at different magnitude changes (Figure 5A left and right panels, respectively). Unsurprisingly, more significant changes in gene expression were observed between different treatment regimens (i.e., PQ Grass conventional or PQ Grass extended vs. placebo) compared to different time points (i.e., V12 or V15 vs. V1). Differential gene expression that was found to be significant across different time points in PQ Grass conventional and extended groups included *CD1A, CD80, IFNG, TNF, STAT4* and *IL1RL1*, which were upregulated, and *IL13, IL4, FOXP3, RIPK4, TGFB1* and *TFRC*, which were downregulated (all, *p* < 0.05; Figure 5B). Differential gene expression analysis further revealed significant upregulation in genes such as *CCL17, IL33, IL9, RORC, TGFB, IL21 and IFNG*, and downregulation in *GATA3, C1QA, TLR4* and *TFRC* in those who received PQ Grass conventional or extended groups compared to placebo (Figure 5C). Genes associated with Treg cells, such as *IL10, FOXP3, EBI3, IL12A and IL27*, were upregulated in both PQ Grass conventional and extended groups compared to placebo, whilst *SATB1* was downregulated. Though these changes in Treg‐associated genes were clear in V12, they were not maintained at V15 (Figure 5D). A similar trend in expression was observed for *IFNG, RIPK4, CCL20*, and *IL21* in both PQ Grass conventional and extended groups compared to placebo.

### 3.8 A Strong Correlation Was Observed in Biomarker Readouts of PQ Grass‐Treated Patients

**FIGURE 6:**
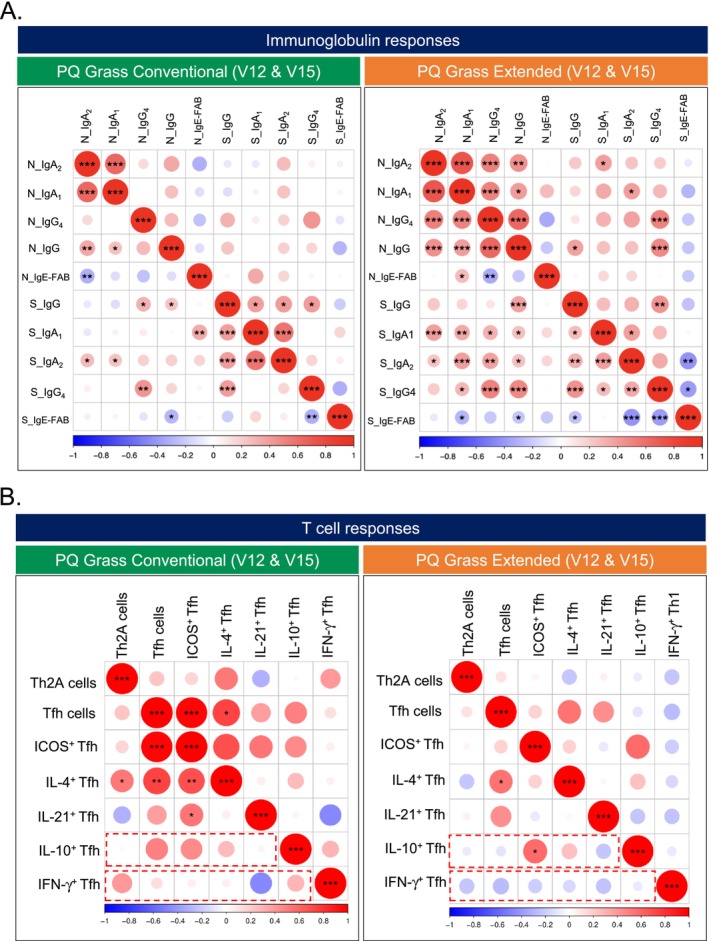
Treatment‐specific correlation analyses. (A) Correlation analyses of immunoglobulin responses in serum and nasal fluid at V12 and V15 in those who received PQ Grass Conventional AIT (left panel) or PQ Grass Extended AIT (right panel). (B) Correlation analyses of proliferated T cell subsets at V12 and V15 in those who received PQ Grass Conventional (left panel) or PQ Grass Extended (right panel). *p < 0.05, **p < 0.01 and ***p < 0.001.

Treatment‐specific correlation analyses between immunoglobulin factors and cellular response was performed to identify any associations between the various biomarkers to predict treatment group and response. Analysis within the immunoglobulin responses in nasal fluid and serum demonstrated significant and strong correlations between various subtypes that is seen in a similar profile for both PQ Grass conventional and extended treatment groups (Figure 6A). Positive correlation was observed between nasal fluid grass‐specific sIgA_1_ and sIgA_2_ (*r* = 0.642, *p* < 0.001), nasal fluid grass‐specific sIgA_2_ and sIgG (*r* = 0.36, *p* < 0.01), serum grass‐specific sIgG and IgA_1_ (*r* = 0.39, *p* < 0.001), serum sIgG and sIgG_4_ (*r* = 0.40, *p* < 0.001) and serum grass‐specific sIgA_1_ and sIgA_2_ (*r* = 0.57, *p* < 0.001) in the PQ Grass conventional group. Similarly, within the PQ Grass extended group, strong correlations were observed amongst different antibody readouts that include nasal fluid grass pollen sIgA_1_ and sIgA_2_ (*r* = 0.57, *p* < 0.001), nasal fluid sIgG_4_ and sIgA_2_ (*r* = 0.71, *p* < 0.001), nasal fluid sIgG_4_ and sIgA_1_ (*r* = 0.52, *p* < 0.001), nasal fluid sIgG and IgG_4_ (*r* = 0.64, *p* < 0.001), serum sIgG and sIgG_4_ (*r* = 0.44, *p* < 0.001), serum sIgA_1_ and sIgA_2_ (*r* = 0.39, *p* < 0.001) and serum sIgG and sIgA_2_ (*r* = 0.31, *p* < 0.01). Most profoundly, a strong correlation between nasal IgE‐FAB blocking response and levels of nasal grass‐specific sIgA_2_ (*r* = −0.31, *p* < 0.01) and nasal sIgG_4_ levels (*r* = −0.34, *p* < 0.01) was observed in PQ Grass conventional and extended groups, respectively. Similarly, at the systemic level, a strong correlation between IgE‐FAB blocking response with serum sIgG_4_ (*r* = −0.32, *p* < 0.01) in PQ Grass conventional and with serum sIgG_4_ (*r* = −0.39, *p* < 0.001) and sIgA_2_ (*r* = −0.42, *p* < 0.001) in PQ Grass extended were observed (Figure 6A).

Interestingly, a differential in correlation can be seen when looking at the cellular compartment for T cells within the PQ Grass conventional and extended treatment groups. Focussing on data obtained post‐treatment at V12 and V15, a strong correlation between various pro‐inflammatory T cells is observed in those who received PQ Grass conventional treatment (Figure 6B). This includes a positive correlation between ICOS^+^ Tfh and Tfh cells (*r* = 0.97, *p* = 0.0001), IL‐4^+^ Tfh and Tfh cells (*r* = 0.74, *p* = 0.0012) and IL‐4^+^ Tfh and Th2A cells (*r* = 0.52, *p* = 0.04). Most importantly, positive correlations are seen between IFN−^+^ Tfh1 and IL‐10^+^ Tfh cells against pro‐inflammatory T cells, which is unexpected (Figure 6B, left panel, red box). In those who received PQ Grass extended treatment, apart from a positive correlation between IL‐4^+^ Tfh and Tfh cells (*r* = 0.54, *p* = 0.03), no significant associations were observed, indicating that one or more T cell subsets are dampened or suppressed (Figure 6B, right panel). More strikingly, albeit not significant, a negative association is observed between IFN‐y^+^ and Th2A cells (*r* = −0.23, *p* = 0.39), Tfh cells (*r* = −0.26, *p* = 0.33), ICOS^+^ Tfh cells (*r* = −0.19, *p* = 0.47) and IL‐4^+^ Tfh cells (*r* = −0.16, *p* = 0.57). With the dampening of Th2 and Tfh cell responses following those who received PQ Grass extended treatment, these observations imply induction of Th1 counterpart, which is favorable following AIT. In line with our flow cytometry observations, correlation analyses demonstrated T cell responses, but not immunoglobulin responses, as key differential features of PQ Grass extended treatment compared to the conventional regimen.

## 4 Discussion

AIT remains to be the only disease‐modifying treatment inducing long‐term clinical benefit for treatment of AR. However, the long‐term treatment regimens being applied, the safety issues observed and, the relatively low treatment compliance rates observed especially with sublingual immunotherapy (SLIT), highlights the need for a safer and more effective treatment with adequate compliance rates. An adjuvant based allergoid immunotherapy, PQ Grass, is classed as a novel short‐course AIT approach for the treatment of grass induced SAR. Allergoids have been shown to maintain sequential IgG and T cell epitopes important for efficacy (B cells induction and T cell activation) whilst disrupting conformational (IgE) epitopes, thus having reduced allergenicity [22, 28, 29, 30, 31, 32, 33, 35, 36, 37]. In this RDBPC exploratory field study, the underlying mechanism of action of two different treatment regimen of a short‐course PQ Grass AIT treatment (i.e., conventional or extended) was investigated. Although a positive clinical efficacy readout was achieved in both treatment arms, a stronger improvement was observed in those who received the extended regimen. The underlying mechanism of action for both conventional and extended AIT were investigated by measuring allergy‐related biomarker readout. Our study revealed no significant differences in the profile of immunoglobulin factors being induced locally or systemically by both conventional and extended AIT, with both treatment regimen inducing similar magnitude of allergen‐specific IgG and IgA antibodies which are functional and can inhibit allergen‐IgE complexes binding to CD23 (FcεRII) on B cells. Gene expression analyses demonstrated similar molecular mechanisms with both treatment arms downregulating Th2‐associated genes and upregulating Treg‐associated genes. However, interrogation of cellular responses, specifically within the T cell compartment, revealed noticeable differences between the two active treatment arms. Compared to conventional AIT, those who received extended AIT displayed a strong trend of dampening in the proliferation of pro‐inflammatory Th2, Th2A and Tfh cells, which was supported by unbiased clustering analyses. Furthermore, those receiving extended AIT also displayed evidence of immune deviation towards Th1 response and the induction of IL‐10^+^ Tfh cells. Interestingly, FOXP3^+^ Treg cells appear to be preferentially induced by conventional AIT. Collectively, though these findings require further validation in a bigger clinical study, our study demonstrates that modulation of T cell compartment following PQ Grass extended AIT may underlie their superior clinical improvement.

In a recent publication, an enhanced clinical efficacy profile of PQ Grass extended regimen for primary and secondary endpoints (CSMS and RQLQ‐S score, respectively) compared to conventional regimen was demonstrated [33]. Primary and secondary endpoints data reported by De Kam et al. [33] was further confirmed in this study by reduction in TCS for both PQ Grass treatment groups compared to placebo groups. However, clinical efficacy does not always correlate with surrogate quantitative endpoints, and to date, there is no consensus on a generally accepted surrogate biomarker, which can be used as a prognostic, predictive and surrogate biomarker of the clinical response [38]. Thus, uncovering the mechanism of action for novel PQ Grass SCIT AIT treatment regimens is of paramount importance not only for identification of a more efficacious therapy for AR but also for addressing unmet needs for surrogate biomarkers of AIT.

Successful AIT is associated with the protective umbrella of allergen‐neutralising specific antibodies with blocking capacity primarily of IgG class (IgG_1_/IgG_4_) [39, 40, 41]. Several studies demonstrated that IgG antibody class is not the only antibody with blocking and allergen‐neutralising functionality and allergen‐specific IgA antibody class (sIgA_1_/sIgA_2_) is equally important [42, 43, 44, 45]. De Kam et al. reported consistently elevated level of grass specific sIgG_4_ in serum [33] for PQ Grass conventional and extended treatment groups for all study population, though this did not translate in statistically significant induction locally in the nasal fluid reported in our study for the sub‐group of subjects who participated in the mechanistic biomarker sub‐study. A more thorough investigation of the immunoglobulin profile between the two PQ Grass treatment regimens revealed similar levels of grass‐specific sIgG, sIgA_1_, and sIgA_2_ for this sub‐group. Assessment of blocking capacity by nasal fluid and serum demonstrated similar functional capacity in the two PQ Grass regimens. Though SCIT regimen can vary depending on the product being administered and the allergen targeted for treatment, the conventional approach involves an up‐dosing period which is followed by a weekly maintenance injection. However, enhanced immunological responses have previously been described following prolonged antigen exposure achieved through a monthly maintenance dose, rather than weekly basis [46]. The PQ Grass extended arm in this study involved a persistent exposure to the sensitising antigen which is thought to be able to modulate long‐term humoral immune responses through the regulation of B cell expansion, plasma cell formation and production of blocking antibodies [47, 48]. Although a differential induction in blocking antibodies have been described previously depending on the type of AIT being given with SCIT preferentially inducing sIgG_4_ whilst SLIT preferentially inducing sIgA_1/2_ [6], importantly, for the first time we were able to demonstrate in this study the induction of grass‐specific sIgG_4_, sIgA_1_ and sIgA_2_ by SCIT PQ Grass at the systemic level.

Long‐term clinical efficacy following AIT is thought to be driven by several key cellular immune responses that involves all aspects of the adaptive immune compartment. This includes dampening of pro‐inflammatory Th2, Th2A and Tfh cells with reduced capacity to produce type 2 cytokines (IL‐4, IL‐5 and IL‐13), immune deviation towards a Th1 response and induction of T and B regulatory cells [38, 44, 49]. In this mechanistic study, we demonstrated a noticeable difference in the mechanisms underlying conventional and extended PQ Grass regimens with the latter being more superior in inducing all these expected immune responses modulated by AIT, except for B regulatory cells. More specifically, PQ Grass extended regimen was superior in it's ability to induce IL‐10^+^ Tfh cells which has not been described previously as a mechanism of tolerance induction by AIT. Tfh cells are relatively novel immune cells shown to be key players in the pathophysiology of allergic inflammation. Recent studies have shown that a subset of Tfh cells, which can produce IL‐10, are proficient in dampening the induction of B cell class‐switching to IgE which can then result in suppression of allergic inflammation [50]. Several factors drive the differentiation, function, and regulation of IL‐10^+^ Tfh cells, which include cytokine and transcriptional regulation and co‐stimulatory molecule. It has been previously described that accrual of IL‐10+ Tfh cells requires Tfh cytokines IL‐6 and IL‐21. These cytokines are critical for the differentiation of conventional Tfh cells, which subsequently influence IL‐10 production. Both IL‐6 and IL‐21 promote the development of Tfh cells by inducing transcription factors like Bcl‐6 and STAT3. More recently, IL‐27 has also been identified as a major inducer of IL‐10 production in T cells, including Tfh cells. IL‐27 signalling through STAT1 and STAT3 pathways leads to IL‐10 production, promoting the differentiation of IL‐10^+^ Tfh cells. Through our gene microarray analyses, it can be seen that both PQ Grass Conventional and Extended treatment upregulated IL12A mRNA whilst IL27 and IL21 was only upregulated in those who receive PQ Grass Extended and not conventional. Though further investigation is required to fully elucidate the molecular mechanism underlying the differential kinetic of induction of IL‐10+ Tfh cells, it is likely that differential kinetic in the transcriptional changes of these genes are a major contributor. Moreover, level of IL‐6 was not analysed during the study and a differential kinetics in the induction of IL‐6 may also be another contributor. Our data highlight that the differential mechanism between the two PQ Grass regimens lie within the cellular T cell responses, rather than the humoral response. Treatment‐specific correlation analyses confirmed these observations in which a shift from a pro‐inflammatory Th2/Tfh response towards a Th1 response is observed in the PQ Grass extended treatment group.

The induction of T regulatory cells within the adaptive immune compartment has been reported following an efficacious AIT across various studies [15, 18, 44, 51]. Treg cells can be further classified into natural and inducible Treg cells, depending on how they are derived [52]. Our study demonstrated that both PQ Grass treatment regimens upregulate Treg‐associated genes that are indicative of the induction of natural and inducible Treg (*FOXP3*), iT_R_1 cells (*IL10*), iT_R_3 cells (*TGFB*) and iT_R_35 cells (*IL12A* and *EBI3*). More importantly, both PQ Grass treatment regimens were associated with the downregulation of the *SATB1* gene, which is important in determining the phenotype and functionality of Treg cells. The *SATB1* gene can be repressed by its master regulator, FOXP3, directly by binding to the *SATB1* locus or indirectly by inducing microRNAs that bind the 3’ UTR end of *SATB1* [53]. When not repressed, *SATB1* expression has been shown to cause a loss of suppressive Treg function and induction of T effector cytokines, which can propagate allergic inflammation. Interestingly, these favourable gene modifications by both conventional and extended PQ Grass were observed primarily at Visit 12 but not retained at Visit 15. To corroborate these findings, we demonstrated that FOXP3^+^ Treg cells were induced at the protein level by both conventional and extended PQ Grass, albeit with a difference in the kinetic response. As extended PQ Grass treatment is associated with a more persistent allergen exposure, it can be speculated that this results in a Treg response that is induced more slowly but sustained for a longer period, which would be ideal in the case of a short‐course AIT treatment in inducing a sustained immune modulation.

Despite the evidence shown in this study, it is noteworthy to acknowledge that many of the cellular observations were based on strong trends, rather than a significant change, due to the small sample size resulting from technical challenges in obtaining the required number of cells for culture. For the same technical reason, we were also unable to investigate the ability of PQ Grass treatment in generating Breg cells. Breg cells have been shown to play a key role in tolerance induction following AIT [54, 55, 56, 57, 58, 59] through several mechanisms that include suppression of T_H_2 cells [20], induction of Treg cells [59], and production of allergen‐neutralising IgG_4_ antibodies [56]. Whilst we were unable to enumerate the proportion of Breg cells following conventional and extended PQ Grass treatment, the generation of blocking antibodies IgG_4_, upregulation of *IL10* and *IL21* at the gene level and maintenance of IL‐21^+^ Tfh cells are all indicative of the likelihood of Breg induction following PQ Grass treatment. Further studies to confirm these trends were warranted and planned in the Phase III trial.

In summary, a short‐course PQ Grass 27,600 SU treatment, conventional or extended, displayed a similar mechanism of action within the humoral compartment. The enhanced efficacy profile of the extended PQ Grass regimen is underscored by stronger modulation of the T cell compartment at the gene and protein level that includes dampening of the Th2 response, immune deviation towards a Th1 response, and induction of IL‐10^+^ Tfh cells. Furthermore, a more gradual and sustained FOXP3^+^ Treg cell induction was observed following the extended PQ Grass regimen, further substantiating the superiority of this regimen. Altogether, our findings provide early evidence of the enhanced tolerogenic cellular and molecular modifications induced by the PQ Grass extended regimen (27,600 SU) in comparison to the PQ Grass conventional regimen.

## Author Contributions

J.A.L., L.Y.D.W., E.P., X.M., S.T.K., P.H., P.F. and A.C.P. performed experiments and analyses of data. W.T.F. performed correlation analysis. J.A.L., M.H.S., S.S., P.‐J.D.K. and M.D.H. designed the biomarker mechanistic study. J.A.L., M.H.S., S.S. and P.‐J.D.K. wrote the manuscript and interpreted the data. P.‐J.D.K., K.O., M.A.S. and M.F.K. designed clinical trial methodology and treatment regimens. K.L., O.A. and S.J.H. managed the active phase of the clinical trial. All authors critically read the manuscript and provided feedback.

## Conflicts of Interest

L.Y.D.W., E.P., X.M., S.T.K., W.T.F.., P.H., P.F., and A.C.P. declare no conflicts of interest. J.A.L. reports grants via Biomedical Research Funding (Imperial College BRC), all outside the submitted work; M.H.S. reports research grants from Immune Tolerance Network, Medical Research Council, Allergy Therapeutics, LETI Laboratorios, Rovolo Biotherapeutics and lecture fees from Allergy Therapeutics and Leti Laboratorios, all outside the submitted work. S.S., P.‐J.D.K., K.O., K.L., O.A., M.A.S., M.D.H., S.J.H., and M.F.K. are former or current employees of Allergy Therapeutics PLC. K.L. and M.F.K. are also current employees of Bencard Allergie GmbH.

## Supporting information


Appendix S1.

Figure S1.

Figure S2.

Figure S3.

## Data Availability

The data that support the findings of this study are available from the corresponding author upon reasonable request.
