# Supplementary material for: Modulation of Cellular, Molecular, and Humoral Responses by PQ Grass 27,600 SU for the Treatment of Seasonal Allergic Rhinitis: A Randomised Double Blind Placebo Control Exploratory Field Study
Source: Allergy. 2025 Jul 8;81(1):232–47. doi: 10.1111/all.16640 (PMC12773655; doi:10.1111/all.16640)
Supplement: Supplementary file 1 — Appendix S1. [file ALL-81-232-s004.docx]

**ONLINE REPOSITORY MATERIALS**

**Modulation of cellular, molecular and humoral responses by PQ Grass 27600 SU for the treatment of seasonal allergic rhinitis: A RDBPC Exploratory Field Study**

Janice A. Layhadi^1*^, Sviatlana Starchenka^2*^, Pieter-Jan De Kam^2*^, Elizabeth Palmer^1^, Lily Y.D. Wu^1^, Sean T. Keane^1^, William Fulton^1^, Prista Hikmawati^1^, Xun Meng^1^, Paulina Filipaviciute^1^, Anna Cutrina^1^, Kemi Oluwayi^2^, Lis Katarzyna^2,3^, Oliver Armfield^2^, Murray A. Skinner^2^, Matthew D. Heath^2^, Simon J Hewings^2^, Matthias F. Kramer^2^, Mohamed H. Shamji^1*^

^1^ National Heart and Lung Institute, Imperial College London, London SW7 2AZ, United Kingdom

^2^ Allergy Therapeutics PLC, Worthing BN14 8SA, United Kingdom

^3^ Bencard Allergie GmbH, Munich 80804, Germany

*Equally contributed to the study

**Corresponding author:**

Professor Mohamed H. Shamji

Immunomodulation and Tolerance Group, Allergy & Clinical Immunology,

Inflammation, Repair and Development, National Heart and Lung Institute,

Sir Alexander Fleming Building, Imperial College London,

South Kensington Campus, London SW7 2AZ, United Kingdom

Tel: +44 020 7594 3476

E-mail: [m.shamji@imperial.ac.uk](mailto:m.shamji@imperial.ac.uk)

**Nasal fluid collection**

To collect nasal fluid, Nasosorption FX-i devices (Hunt Developments, UK; NSFL-FXI-11) containing a synthetic absorptive matrix was inserted into each subject’s right and left nostrils for 50 seconds to absorb mucosal lining fluid from within the nasal cavity. Nasosorption FX-i devices were weighed and then cryopreserved in liquid nitrogen and shipped at the end of the study to the Immune Tolerance Group research laboratories at Imperial College London. For quantitative and atraumatic processing of nasal secretions, Nasosorption FX-i devices were transferred into Costar*TM* Spin-X*TM* centrifuge tubes containing a membrane 0.22mM filter unit to remove bacteria, cells, and particles from liquids (Corning, 10104101). Next, 300μL of precooled 4°C sterile elution buffer (Merck Millipore, AB-33K) was added directly onto the membrane within the filter tube and then all samples were vortexed and then immediately centrifuged (16000g, 20 minutes, 4°C). Filter inserts containing Nasosorption FX-i devices were discarded and the nasal fluid from the right and left nostril was then pooled whilst on ice and weighed. Processed nasal fluid samples were placed in Protein LoBind Tubes (Eppendorf, 0030108116) and stored at -80°C until downstream immunoglobulin analysis utilising ImmunoCAP or enzyme-linked immunosorbent assays.

**Measurement of grass pollen specific IgE and IgG_4_ in nasal fluid**

Level of grass pollen specific IgE and IgG_4_ in nasal fluid were measured using ImmunoCAP 100 (Phadia & ThermoFisher, Sweden) following the manufacturer’s instructions.

**Measurement of grass pollen-specific IgG, IgA_1_ and IgA_2_**

Level of grass pollen-specific IgG, IgA_1_ and IgA_2_ were measured in nasal fluid, extracted from Nasosortion FX-I devices (Hunt Developments, UK; NSFL-FXI-11) (see supplementary methods) and serum using enzyme linked immunosorbent assay (ELISA). Plates were coated with Timothy grass pollen extract (1 μg/mL for IgA_1_ and IgG, and 5 μg/mL for IgA2) for 1 hour at 37^o^C. Wells were washed three times using wash buffer (0.05% Tween 20 in PBS), dried and blocked using Chonblock buffer (Chondrex) for 1 hour at 37^o^C. Plates were washed with wash buffer and dried completely. Standards and serum or nasal fluid samples were added into corresponding wells and incubated overnight at 4^o^C. Following incubation, wells were washed, dried, and incubated with monoclonal antibodies to IgA_1_, IgA_2_ or IgG (Bio-Rad) for 30 minutes. Finally, wells were incubated with HRP-conjugated streptavidin (Biolegend) for 30 minutes on a shaker before being developed using TMB substrate. Stop solution (2N H_2_SO_4_) was added at the end to stop the reaction. Plates were read at OD_450_ using an ELISA microplate reader (Molecular Probes, Eugene, OR, USA).

**IgE facilitated allergen binding assay**

IgE-FAB to B cells was performed using in-house serum from grass pollen-sensitised donor (sIgE to Phlp > 100 kU_A_/L). Serum was incubated with 0, 0.3 and 1 mg/mL of Timothy grass pollen extract for 1 hour at 37^o^C with and without serum or nasal fluid collected from participants at V1, V12 and V15. EBV-transformed B cells (1x10^5^ cells per test) were added for 1 hour at 4^o^C and washed. Binding of allergen-IgE complexes to the CD23 receptor on the surfaces of B cells was detected by using phycoerythrin-labelled anti-human IgE antibodies (Miltenyi Biotec) and acquired on the BD FACSCanto II (BD Biosciences). Data were analysed using FACSDiva software by quantifying the proportion of IgE^+^ B cells (BD Biosciences).

***In vitro* T cell stimulation**

Peripheral blood mononuclear cells (PBMCs) collected at V1, V12 and V15 were stained with CellTrace Violet (ThermoFisher) and stimulated with 0, 0.03, 0.3, 1 and 5 μg/mL of Timothy grass pollen extract, for 6 days in a 37^o^C incubator, 5% CO_2_. Cells were washed with culture medium and stimulated with phorbol 12-myristate 13-acetate (50 ng/mL, Sigma-Aldrich, UK) and ionomycin (1 μg/mL, Sigma-Aldrich) in the presence of monensin (20 μg/mL, Biolegend) for 5 hours prior to staining. Cells were blocked with Fc blocking agent (Miltenyi Biotec) and immunostained with cell surface markers (CD4, CXCR5, PD-1, ICOS; all from BD Biosciences, and CRTH2, CD27, CD127, CD49d, CD161, CD25; all from Biolegend) and intracellular markers (IL-4, IFN-γ; BD Biosciences, and IL-21, IL-10; Biolegend). Cell proliferation was assessed by CellTrace Violet staining (Life Technologies). Immunostained cells were then acquired on BD LSRFortessa™ where a minimum of 300 events were recorded for each population of interest. Each subset of cells were defined with the following phenotype: Th2 (CD4^+^CD27^-^CRTh2^+^), Th2A (CD4^+^CD27^-^CRTh2^+^CD161^+^CD49d^+^), Tfh (CD4^+^CXCR5^+^PD-1^+^), ICOS^+^ Tfh (ICOS^+^CD4^+^CXCR5^+^PD-1^+^), IL-4^+^ Tfh (IL-4^+^CD4^+^CXCR5^+^PD-1^+^), IL-21^+^ Tfh (IL-21^+^CD4^+^CXCR5^+^PD-1^+^), IL-10^+^ Tfh (IL-10^+^ CD4^+^CXCR5^+^PD-1^+^), and Th1 (CD4^+^IFN-γ^+^). The sensitivity of the flow cytometer readout is generally accepted to be 0.005% of the parent population and therefore the lowest detection limit. Any readout below the lowest detection limit is considered as undetectable.

**Induction of T regulatory cells**

PBMCs collected at V1, V12 and V15 were blocked with Fc blocking agent (Miltenyi Biotec) and immunostained with cell surface markers (CD4, CD25, CD127, CTLA-4, CD45RO, all from Biolegend) and nuclear factor markers (FOXP3 and SATB1, all from BD Biosciences). Immunostained cells were acquired on BD LSRFortessa™ where a minimum of 300 events were recorded for each population of interest. Each subset of cells were defined with the following phenotype: total FOXP3^+^Tregs (CD4^+^CD25^+^CD127^-^FOXP3^+^), memory Tregs (CD4^+^CD25^+^CD127^-^CD45RO^+^FOXP3^+^) and naïve Tregs (CD4^+^CD25^+^CD127^-^CD45RO^-^FOXP3^+^). The sensitivity of the flow cytometer readout is generally accepted to be 0.005% of the parent population and therefore the lowest detection limit. Any readout below the lowest detection limit is considered as undetectable.

**Unbiased clustering analysis**

Unsupervised machine learning analyses were performed on concatenated FCS files from at least 6 independent samples. Clustering analysis with viSNE were performed for the data generated from the immunogenicity assays. viSNE employs t-SNE algorithm that maps individual cells in a 2D or 3D map based on their high dimensional relationship. Clustering of samples were run with equal sampling size to ensure that each cell population represented on the viSNE map is of the same number of cells. In addition to analysis using viSNE, the data were also clustered using FlowSOM, which employs self-organising maps (SOM) to cluster data and visualise the results as a minimum spanning tree. FlowSOM clustering was setup for a defined number of 23 metaclusters of cells across all samples of T cells.

**Microarray gene expression analysis**

A subgroup of 30 subjects randomly selected at two of the European clinical sites consented for the optional biomarker analysis. PBMCs from visits 1, 12 and 15 were collected and RT^2^ profiler PCR array gene expression analysis was performed using RT^2^SYBR^®^ Green ROX qPCR master mix and RT^2^ Profiler PCR array platform (Qiagen, Germany). Expression level was calculated using ΔΔCt and the relative fold change between baseline visit 1 and treatment visits was determined as 2^-ΔΔCt^. Genes with a fold change difference of greater than or equal to 1.2 and *p*-value <0.05 were considered significantly differentially expressed.

**Table S1. Demographics and baseline characteristics.**

| **Variables** | **PQ Grass conventional regimen n=41** | **PQ Grass extended regimen n=40** | **Placebo with MCT n=20** | **Placebo  n=18** | **Total n=119** |
| --- | --- | --- | --- | --- | --- |
| **Age at Screening [years]** |  | | | | |
| Mean (SD) | 34.5 (11.45) | 32.3 (10.23) | 34.3 (10.69) | 31.5 (13.79) | 33.3 (11.24) |
| **Sex [n (%)]** |  | | | | |
| Male | 13 (31.7%) | 19 (47.5%) | 11 (55.0%) | 8 (44.4%) | 51 (42.9%) |
| Female | 28 (68.3%) | 21 (52.5%) | 9 (45.0%) | 10 (55.6%) | 68 (57.1%) |
| **Height [cm] at Screening** |  | | | | |
| Mean (SD) | 170.86 (10.365) | 173.25 (9.072) | 174.20 (7.161) | 171.59 (10.958) | 172.33 (9.533) |
| **Weight [kg] at Screening** |  | | | | |
| Mean (SD) | 76.30 (15.092) | 77.71 (16.336) | 76.95 (18.903) | 74.71 (11.815) | 76.64 (15.623) |
| **Allergic Rhinitis** |  | | | | |
| n (%) | 41 (100.0%) | 40 (100.0%) | 20 (100.0%) | 18 (100.0%) | 119 (100.0%) |
| Severe | 20 (48.8%) | 12 (30.0%) | 9 (45.0%) | 8 (44.4%) | 49 (41.2%) |
| Moderate | 21 (51.2%) | 28 (70.0%) | 11 (55.0%) | 10 (55.6%) | 70 (58.8%) |
| Mild | 0 (0.0%) | 0 (0.0%) | 0 (0.0%) | 0 (0.0%) | 0 (0.0%) |
| **Allergic Conjunctivitis** |  | | | | |
| n (%) | 35 (85.4%) | 31 (77.5%) | 16 (80.0%) | 14 (77.8%) | 96 (80.7%) |
| Severe | 17 (41.5%) | 11 (27.5%) | 3 (15.0%) | 5 (27.8%) | 36 (30.3%) |
| Moderate | 18 (43.9%) | 19 (47.5%) | 11 (55.0%) | 9 (50.0%) | 57 (47.9%) |
| Mild | 0 (0.0%) | 1 (2.5%) | 2 (10.0%) | 0 (0.0%) | 3 (2.5%) |
| **Allergic Cough** |  | | | | |
| n (%) | 5 (12.2%) | 8 (20.0%) | 5 (25.0%) | 3 (16.7%) | 21 (17.6%) |
| Severe | 1 (2.4%) | 0 (0.0%) | 1 (5.0%) | 0 (0.0%) | 2 (1.7%) |
| Moderate | 1 (2.4%) | 3 (7.5%) | 0 (0.0%) | 2 (11.1%) | 6 (5.0%) |
| Mild | 3 (7.3%) | 5 (12.5%) | 4 (20.0%) | 1 (5.6%) | 13 (10.9%) |
| **Allergic Asthma** |  | | | | |
|  | 6 (14.6%) | 14 (35.0%) | 4 (20.0%) | 4 (22.2%) | 28 (23.5%) |
| Severe | 0 (0.0%) | 0 (0.0%) | 0 (0.0%) | 0 (0.0%) | 0 (0.0%) |
| Moderate | 0 (0.0%) | 2 (5.0%) | 1 (5.0%) | 1 (5.6%) | 4 (3.4%) |
| Mild | 6 (14.6%) | 12 (30.0%) | 3 (15.0%) | 3 (16.7%) | 24 (20.2%) |
| **Allergic Urticaria** |  | | | | |
| n (%) | 7 (17.1%) | 3 (7.5%) | 3 (15.0%) | 3 (16.7%) | 16 (13.4%) |
| Severe | 0 (0.0%) | 0 (0.0%) | 0 (0.0%) | 1 (5.6%) | 1 (0.8%) |
| Moderate | 4 (9.8%) | 1 (2.5%) | 0 (0.0%) | 2 (11.1%) | 7 (5.9%) |
| Mild | 3 (7.3%) | 2 (5.0%) | 3 (15.0%) | 0 (0.0%) | 8 (6.7%) |
| Skin Prick Test* (12 grass mix**) |  |  |  |  |  |
| n (%) | 41 (100.0%) | 40 (100.0%) | 20 (100.0%) | 18 (100.0%) | 119 (100.0%) |
| **Serum grass-specific IgE [kU_A_/L]***** |  | | | | |
| Mean (SD) | 24.9 (31.46) | 31.0 (32.53) | 30.6 (34.39) | 12.8 (12.43) | - |
| Median (Q1, Q3) | 10.2 (4.5, 31.7) | 18.4 (4.8, 48.5) | - 1. (3.9, 47.1) | 9.0 (2.7, 20.6) | - |

- Skin Prick Test positive if wheal (longest diameter) is ≥ 3mm

** 12 grass mix standardized allergen mix from *Pooideae spp.* was used for SPT (*Cynosurus cristatus, Holcus lanatus, Bromus spp., Arrhenatherum elatius, Alopecurus pratensis, Phleum pratense, Festuca pratensis, Lolium perenne/multiflorum, Dactylis glomerata, Poa pratensis/trivialis, Anthoxanthum odoratum, Agrostis tenuis/capillaris*

*** Mix of five grasses from *Pooideae spp.* was used for grass-specific IgE test (ImmunoCap) (*Dactylis glomerata Festuca pratense Lolium perenne Phleum pratense Poa pratensis)*

**Note:** demographics and baseline characteristics are presented for entire clinical subjects population (n=119) based on FAS.

**Abbreviations: FAS = full analysis set;** MCT = MicroCrystalline Tyrosine; SD = standard deviation; SPT = skin prick test

**Table S2. Demographics and baseline characteristics cellular/gene microarray biomarker sub-study.**

| **Variables** | **PQ Grass conventional regimen n=10** | **PQ Grass extended regimen n=10** | **Placebo with MCT n=5** | **Placebo  n=5** | **Total n=30** |
| --- | --- | --- | --- | --- | --- |
| **Age at Screening [years]** |  | | | | |
| Mean (SD) | 31.8 (9.10) | 27.4 (7.65) | 28.4 (4.16) | 27.0 (6.16) | 29.0 (7.47) |
| **Sex [n (%)]** |  | | | | |
| Male | 6 (60.0%) | 6 (60.0%) | 3 (60.0%) | 3 (60.0%) | 18 (60.0%) |
| Female | 4 (40.0%) | 4 (40.0%) | 2 (40.0%) | 2 (40.0%) | 12 (40.0%) |
| **Height [cm] at Screening** |  | | | | |
| Mean (SD) | 175.20 (11.116) | 174.30 (9.034) | 174.80 (5.020) | 175.20 (10.281) | 174.83 (9.432) |
| **Weight [kg] at Screening** |  | | | | |
| Mean (SD) | 74.60 (14.924) | 75.80 (10.737) | 65.40 (4.879) | 71.20 (16.873) | 72.90 (12.710) |
| **Allergic Rhinitis** |  | | | | |
| n (%) | 10 (100.0%) | 9 (90.0%) | 5 (100.0%) | 5 (100.0%) | 29 (96.7%) |
| Severe | 3 (30.0%) | 2 (20.0%) | 3 (60.0%) | 1 (20.0%) | 9 (30.0%) |
| Moderate | 7 (70.0%) | 7 (70.0%) | 2 (40.0%) | 4 (80.0%) | 20 (66.7%) |
| Mild | 0 (0.0%) | 0 (0.0%) | 0 (0.0%) | 0 (0.0%) | 0 (0.0%) |
| **Allergic Conjunctivitis** |  | | | | |
| n (%) | 7 (70.0%) | 6 (60.0%) | 4 (80.0%) | 2 (40.0%) | 19 (63.3%) |
| Severe | 3 (30.0%) | 2 (20.0%) | 1 (20.0%) | 0 (0.0%) | 6 (20.0%) |
| Moderate | 4 (40.0%) | 4 (40.0%) | 3 (60.0%) | 2 (40.0%) | 13 (43.3%) |
| Mild | 0 (0.0%) | 0 (0.0%) | 0 (0.0%) | 0 (0.0%) | 0 (0.0%) |
| **Allergic Cough** |  | | | | |
| n (%) | 2 (20.0%) | 1 (10.0%) | 3 (60.0%) | 0 (0.0%) | 6 (20.0%) |
| Severe | 0 (0.0%) | 0 (0.0%) | 1 (20.0%) | 0 (0.0%) | 1 (3.3%) |
| Moderate | 1 (10.0%) | 0 (0.0%) | 0 (0.0%) | 0 (0.0%) | 1 (3.3%) |
| Mild | 1 (10.0%) | 1 (0.0%) | 2 (40.0%) | 0 (0.0%) | 4 (13.3%) |
| **Allergic Asthma** |  | | | | |
|  | 3 (30.0%) | 4 (40.0%) | 3 (60.0%) | 2 (40.0%) | 12 (40.0%) |
| Severe | 0 (0.0%) | 0 (0.0%) | 0 (0.0%) | 0 (0.0%) | 0 (0.0%) |
| Moderate | 0 (0.0%) | 1 (10.0%) | 1 (20.0%) | 0 (0.0%) | 2 (6.7%) |
| Mild | 3 (30.0%) | 3 (30.0%) | 2 (40.0%) | 2 (40.0%) | 10 (33.3%) |
| Skin Prick Test* (12 grass mix**) |  |  |  |  |  |
| n (%) | 10 (100.0%) | 10 (100.0%) | 5 (100.0%) | 5 (100.0%) | 30(100.0%) |
| **Serum grass-specific IgE [kU_A_/L]***** |  | | | | |
| Mean (SD) | 46.4 (40.43) | 23.0 (22.07) | 53.1 (46.82) | 17.3 (19.54) | 34.9 (34.91) |
| Median (Q1, Q3) | 43.1 (5.7, 84.7) | - 1. (11.6, 26.2) | 67.2 (3.5, 95.6) | 3.7 (3.3, 38.0) | 23.0 (3.68, 71.25) |

- Skin Prick Test positive if wheal (longest diameter) is ≥ 3mm

** 12 grass mix standardized allergen mix from *Pooideae spp.* was used for SPT (*Cynosurus cristatus, Holcus lanatus, Bromus spp., Arrhenatherum elatius, Alopecurus pratensis, Phleum pratense, Festuca pratensis, Lolium perenne/multiflorum, Dactylis glomerata, Poa pratensis/trivialis, Anthoxanthum odoratum, Agrostis tenuis/capillaris*

*** Mix of five grasses from *Pooideae spp.* was used for grass-specific IgE test (ImmunoCap) (*Dactylis glomerata Festuca pratense Lolium perenne Phleum pratense Poa pratensis)*

**Note:** demographics and baseline characteristics are presented for biomarker sub-study group (n=30) based on FAS.

**Table S3. Total Combined Score (TCS) for all population of PQG309**

| **Endpoint** | **% difference effect size (80% CI)** | |
| --- | --- | --- |
|  | **Conventional AIT regimen (n=41)** | **Extended AIT regimen**  **(n=40)** |
| **PQ Grass vs Placebo + MCT** |  |  |
| TCS during peak GPS | -35.0% (-51.2%; -18.9%) | -40.8% (-56.4%; -25.1%) |
| TCS-dSS during peak GPS | -26.1% (-41.7%; 10.5%) | -29.6% (-44.9%; -14.3%) |
| TCS-dMS during peak GPS | -47.0% (-67.4%; -26.7%) | -55.8% (-75.4%; -36.2%) |
| **PQ Grass vs Placebo** |  |  |
| TCS during peak GPS | -19.1% (-42.6%; 4.4%) | -26.2% (-48.9%; -3.5%) |
| TCS-dSS during peak GPS | -20.9% (-38.9%; -2.9%) | -24.6% (-42.3%; -7.0%) |
| TCS-dMS during peak GPS | -15.4% (-57.8%; 27.0%) | -29.4% (-68.6%; 9.8%) |

**Abbreviations:** CI = confidence interval; GPS = grass pollen season; dMS = daily medication score; dSS = daily symptom score**;** MCT = MicroCrystalline Tyrosine; TCS = total combined score.

**Table S4. Total Combined Scores (TCS) for the biomarker subgroup of PQG309**

| Endpoint | % difference effect size (80% CI) | |
| --- | --- | --- |
|  | Conventional AIT regimen (n=10) | Extended AIT regimen (n=10) |
| **PQ Grass vs Placebo + MCT** |  |  |
| TCS during peak GPS | -29.7% (-64.1%; 4.7%) | -46.9% (-77.1%; -16.7%) |
| TCS-dSS during peak GPS | -21.0% (-54.2%; 12.2%) | -36.0% (-65.5%; -6.6%) |
| TCS-dMS during peak GPS | -42.2% (-85.3%; 0.9%) | -62.4% (-100.7%; -24.2%) |
| **PQ Grass vs Placebo** |  |  |
| TCS during peak GPS | -31.9% (-65.1%; 1.3%) | -48.6% (-77.4%; -19.7%) |
| TCS-dSS during peak GPS | -27.0% (-56.8%; 2.9%) | -40.9% (-67.2%; -14.6%) |
| TCS-dMS during peak GPS | -39.8% (-85.7%; 6.0%) | -60.9% (-101.1%; -20.7%) |

**Abbreviations:** CI = confidence interval; dMS = daily medication score; dSS = daily symptom score**;** MCT = MicroCrystalline Tyrosine; TCS = total combined score.

**Table S5. Statistical analysis for the level of serum sIgG between treatment groups (PQ Grass Conventional (n=25), PQ Grass Extended (n=26), Placebo with MCT (n=13) and Placebo (saline; n=12)**

|  |  | Difference in median | Adjusted P value |
| --- | --- | --- | --- |
| VISIT 12 | Conventional AIT regimen vs. Extended AIT regimen | 0.79 | 0.8239 |
|  | **Conventional AIT regimen vs. Placebo + MCT** | **-30.70** | **0.0119 *** |
|  | **Conventional AIT regimen vs. Placebo** | **-38.71** | **0.0010 ***** |
|  | **Extended AIT regimen vs. Placebo + MCT** | **-31.49** | **0.0059 **** |
|  | **Extended AIT regimen vs. Placebo** | **-39.50** | **0.0004 ***** |
| VISIT 15 | Conventional AIT regimen vs. Extended AIT regimen | 19.43 | 0.2846 |
|  | **Conventional AIT regimen vs. Placebo + MCT** | **-34.61** | **0.0262 *** |
|  | **Conventional AIT regimen vs. Placebo** | **-40.02** | **0.0052 **** |
|  | **Extended AIT regimen vs. Placebo + MCT** | **-54.03** | **0.0037 **** |
|  | **Extended AIT regimen vs. Placebo** | **-59.44** | **0.0003 ***** |

**Abbreviations:** AIT = allergen immunotherapy; MCT = MicroCrystalline Tyrosine.

* P<0.05, ** P<0.01 and *** P<0.001.

**Table S6. Statistical analysis for the level of serum sIgA_1_ between treatment groups (PQ Grass Conventional (n=25), PQ Grass Extended (n=26), Placebo with MCT (n=13) and Placebo (saline; n=12)**

|  |  | Difference in median | Adjusted P value |
| --- | --- | --- | --- |
| VISIT 12 | Conventional AIT regimen vs. Extended AIT regimen | -3.870 | 0.5278 |
|  | **Conventional AIT regimen vs. Placebo + MCT** | **-13.64** | **0.0118 *** |
|  | Conventional AIT regimen vs. Placebo | -8.380 | 0.2172 |
|  | **Extended AIT regimen vs. Placebo + MCT** | **-9.770** | **0.0408 *** |
|  | Extended AIT regimen vs. Placebo | -4.510 | 0.3426 |
| VISIT 15 | Conventional AIT regimen vs. Extended AIT regimen | 5.600 | 0.6561 |
|  | **Conventional AIT regimen vs. Placebo + MCT** | **-4.840** | **0.0288 *** |
|  | Conventional AIT regimen vs. Placebo | -0.200 | 0.8840 |
|  | **Extended AIT regimen vs. Placebo + MCT** | **-10.44** | **0.0139 *** |
|  | Extended AIT regimen vs. Placebo | -5.800 | 0.6945 |

**Abbreviations:** AIT = allergen immunotherapy; MCT = MicroCrystalline Tyrosine.

* P<0.05, ** P<0.01 and *** P<0.001.

**Table S7. Statistical analysis for the level of serum sIgA_2_ between treatment groups (PQ Grass Conventional (n=25), PQ Grass Extended (n=26), Placebo with MCT (n=13) and Placebo (saline; n=12)**

|  |  | Difference in median | Adjusted P value |
| --- | --- | --- | --- |
| VISIT 12 | Conventional AIT regimen vs. Extended AIT regimen | 3.950 | 0.6742 |
|  | **Conventional AIT regimen vs. Placebo + MCT** | **-19.43** | **0.0013 **** |
|  | **Conventional AIT regimen vs. Placebo** | **-16.62** | **0.0017 **** |
|  | **Extended AIT regimen vs. Placebo + MCT** | **-23.38** | **0.0031 **** |
|  | **Extended AIT regimen vs. Placebo** | **-20.57** | **0.0031 **** |
| VISIT 15 | Conventional AIT regimen vs. Extended AIT regimen | 4.570 | 0.3650 |
|  | Conventional AIT regimen vs. Placebo + MCT | -13.84 | 0.1035 |
|  | **Conventional AIT regimen vs. Placebo** | **-8.520** | **0.0381 *** |
|  | Extended AIT regimen vs. Placebo + MCT | -18.41 | 0.1251 |
|  | **Extended AIT regimen vs. Placebo** | **-13.09** | **0.0270 *** |

**Abbreviations:** AIT = allergen immunotherapy; MCT = MicroCrystalline Tyrosine.

* P<0.05, ** P<0.01 and *** P<0.001.

**Table S8. Statistical analysis for the relative allergen-IgE binding to B cells across different treatment groups (PQ Grass Conventional (n=25), PQ Grass Extended (n=26), Placebo with MCT (n=13) and Placebo (saline; n=12)**

|  |  |  | Difference in median | Adjusted P value |
| --- | --- | --- | --- | --- |
| NASAL FLUID | VISIT 12 | Conventional AIT regimen vs. Extended AIT regimen | 4.045 | 0.6544 |
|  |  | **Conventional AIT regimen vs. Placebo + MCT** | **15.23** | **0.0027 **** |
|  |  | Conventional AIT regimen vs. Placebo | 8.780 | 0.1310 |
|  |  | **Extended AIT regimen vs. Placebo + MCT** | **11.19** | **0.011 *** |
|  |  | Extended AIT regimen vs. Placebo | 4.735 | 0.2115 |
|  | VISIT 15 | Conventional AIT regimen vs. Extended AIT regimen | -0.475 | 0.6407 |
|  |  | **Conventional AIT regimen vs. Placebo + MCT** | **11.81** | **0.0401 *** |
|  |  | Conventional AIT regimen vs. Placebo | 7.980 | 0.0932 |
|  |  | Extended AIT regimen vs. Placebo + MCT | 12.29 | 0.1162 |
|  |  | Extended AIT regimen vs. Placebo | 8.455 | 0.1042 |
| SERUM | VISIT 12 | Conventional AIT regimen vs. Extended AIT regimen | 1.985 | 0.9447 |
|  |  | **Conventional AIT regimen vs. Placebo + MCT** | **25.26** | **0.0279 *** |
|  |  | Conventional AIT regimen vs. Placebo | 17.67 | 0.1042 |
|  |  | **Extended AIT regimen vs. Placebo + MCT** | **23.27** | **0.0127 *** |
|  |  | **Extended AIT regimen vs. Placebo** | **15.68** | **0.0449 *** |
|  | VISIT 15 | Conventional AIT regimen vs. Extended AIT regimen | -5.840 | 0.8043 |
|  |  | **Conventional AIT regimen vs. Placebo + MCT** | **9.170** | **0.0359 *** |
|  |  | Conventional AIT regimen vs. Placebo | 8.055 | 0.118 |
|  |  | **Extended AIT regimen vs. Placebo + MCT** | **15.01** | **0.0352 *** |
|  |  | Extended AIT regimen vs. Placebo | 13.90 | 0.0615 |

**Abbreviations:** AIT = allergen immunotherapy; MCT = MicroCrystalline Tyrosine.

* P<0.05, ** P<0.01 and *** P<0.001.

**Table S9. Descriptive statistics for raw proliferated Th2, Th2A and Tfh cell responses (PQ Grass Conventional (n=10), PQ Grass Extended (n=10), Placebo with MCT (n=5) and Placebo (saline; n=5)**

**SUPPLEMENTAL FIGURE LEGENDS**

**Figure S1. Subjects disposition flow diagram.** A of 119 subjects were randomized into four treatment arms: PQ Grass conventional (n=41), PQ Grass extended (n=40), placebo with MCT (n=20), placebo (n=18). 114 subjects completed the study.

**Figure S2.** Relative difference in Total Combined Scores (TCS), TCS-dSS, TCS-dMS of PQ Grass conventional or extended AIT groups compared to placebo.

**Figure S3. Extended PQ Grass regimen has the capacity to suppress proliferation of pro-inflammatory T cell responses.** (A-C) Percentage of proliferation of *(A)* Th2 *(B)* Th2A and *(C)* Tfh cells in the presence of increasing concentrations for grass-pollen extract (*P. pratense)* for both PQ Grass conventional and extended AIT groups and the placebo groups for each visit 1, 12 and 15. where n = 10, 10, 5 and 5 for PQ Grass conventional, PQ Grass extended, placebo with MCT and placebo regimens, respectively.

**SUPPLEMENTAL TABLES**

**Table S1. Demographics and baseline characteristics.**

**Table S2: Demographics and baseline characteristics mechanistic (biomarker sub-study).**

**Table S3. Descriptive Statistics for Total Combined Scores (TCS) for all population of PQG309 study**

**Table S4. Total Combined Scores (TCS) for the biomarker subgroup of PQG309**

**Table S5. Statistical analysis for the level of serum sIgG between treatment groups (PQ Grass Conventional (n=25), PQ Grass Extended (n=26), Placebo with MCT (n=13) and Placebo (saline; n=12)**

**Table S6. Statistical analysis for the level of serum sIgA1 between treatment groups (PQ Grass Conventional (n=25), PQ Grass Extended (n=26), Placebo with MCT (n=13) and Placebo (saline; n=12)**

**Table S7. Statistical analysis for the level of serum sIgA2 between treatment groups (PQ Grass Conventional (n=25), PQ Grass Extended (n=26), Placebo with MCT (n=13) and Placebo (saline; n=12)**

**Table S8. Statistical analysis for the relative allergen-IgE binding to B cells across different treatment groups (PQ Grass Conventional (n=25), PQ Grass Extended (n=26), Placebo with MCT (n=13) and Placebo (saline; n=12)**

**Table S9. Descriptive statistics for raw proliferated Th2, Th2A and Tfh cell responses (PQ Grass Conventional (n=10), PQ Grass Extended (n=10), Placebo with MCT (n=5) and Placebo (saline; n=5)**
