# Supplementary material for: Modulation of Cellular, Molecular, and Humoral Responses by PQ Grass 27,600 SU for the Treatment of Seasonal Allergic Rhinitis: A Randomised Double Blind Placebo Control Exploratory Field Study
Source: Allergy. 2025 Jul 8;81(1):232–47. doi: 10.1111/all.16640 (PMC12773655; doi:10.1111/all.16640)
Supplement: Supplementary file 2 — Figure S1. [file ALL-81-232-s001.pptx]

## Slide 1
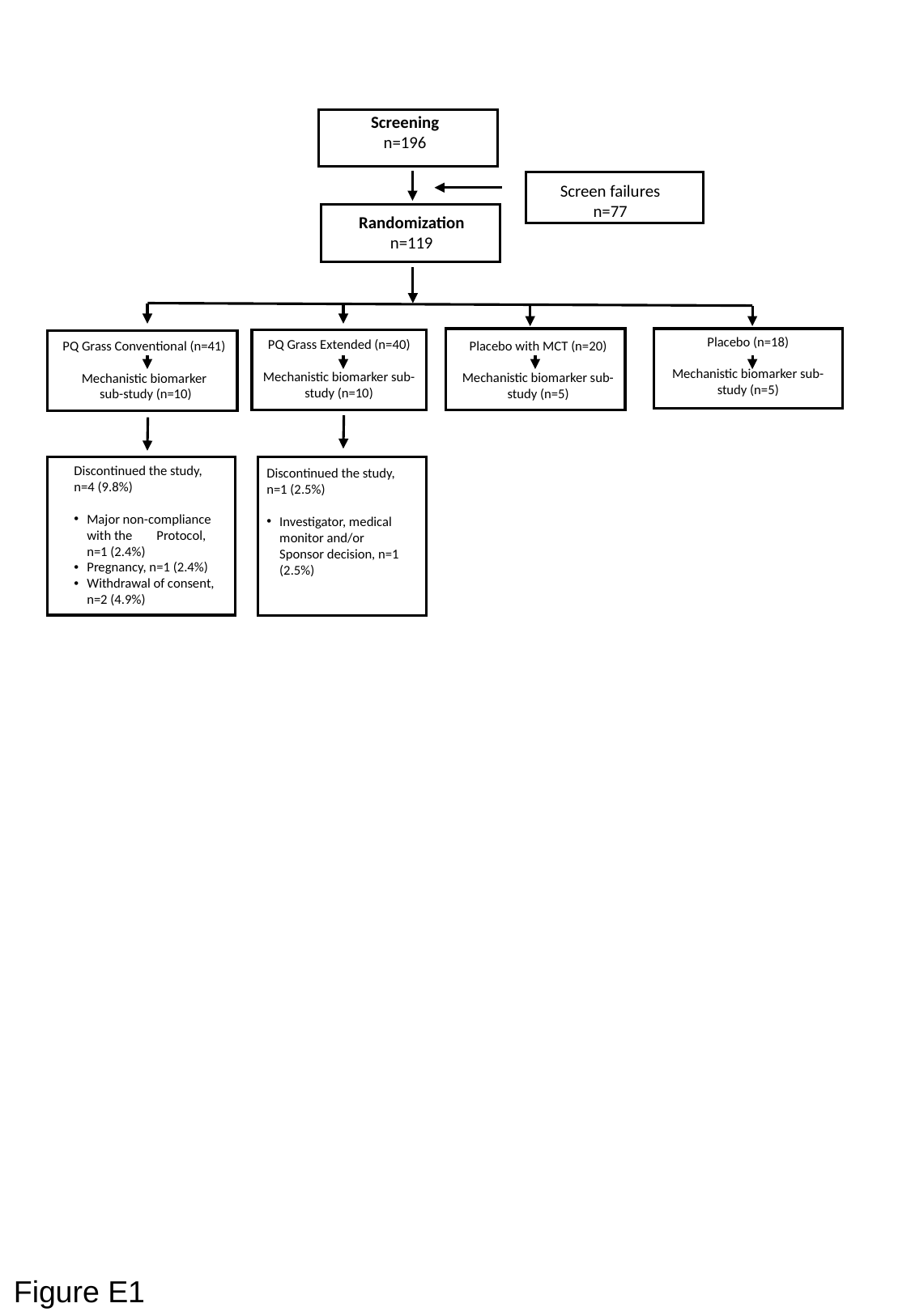

Screening
n=196
Screen failures
n=77
Randomization
n=119
Placebo (n=18)
Mechanistic biomarker sub-study (n=5)
PQ Grass Extended (n=40)
Mechanistic biomarker sub-study (n=10)
PQ Grass Conventional (n=41)
Mechanistic biomarker
 sub-study (n=10)
Placebo with MCT (n=20)
Mechanistic biomarker sub-study (n=5)
Discontinued the study, n=4 (9.8%)
Major non-compliance with the Protocol, n=1 (2.4%)
Pregnancy, n=1 (2.4%)
Withdrawal of consent, n=2 (4.9%)
Discontinued the study, n=1 (2.5%)
Investigator, medical monitor and/or Sponsor decision, n=1 (2.5%)
Figure E1
