# Supplementary figures and images for: Modulation of Cellular, Molecular, and Humoral Responses by PQ Grass 27,600 SU for the Treatment of Seasonal Allergic Rhinitis: A Randomised Double Blind Placebo Control Exploratory Field Study
Source: Allergy. 2025 Jul 8;81(1):232–47. doi: 10.1111/all.16640 (PMC12773655; doi:10.1111/all.16640)

## Slide 1
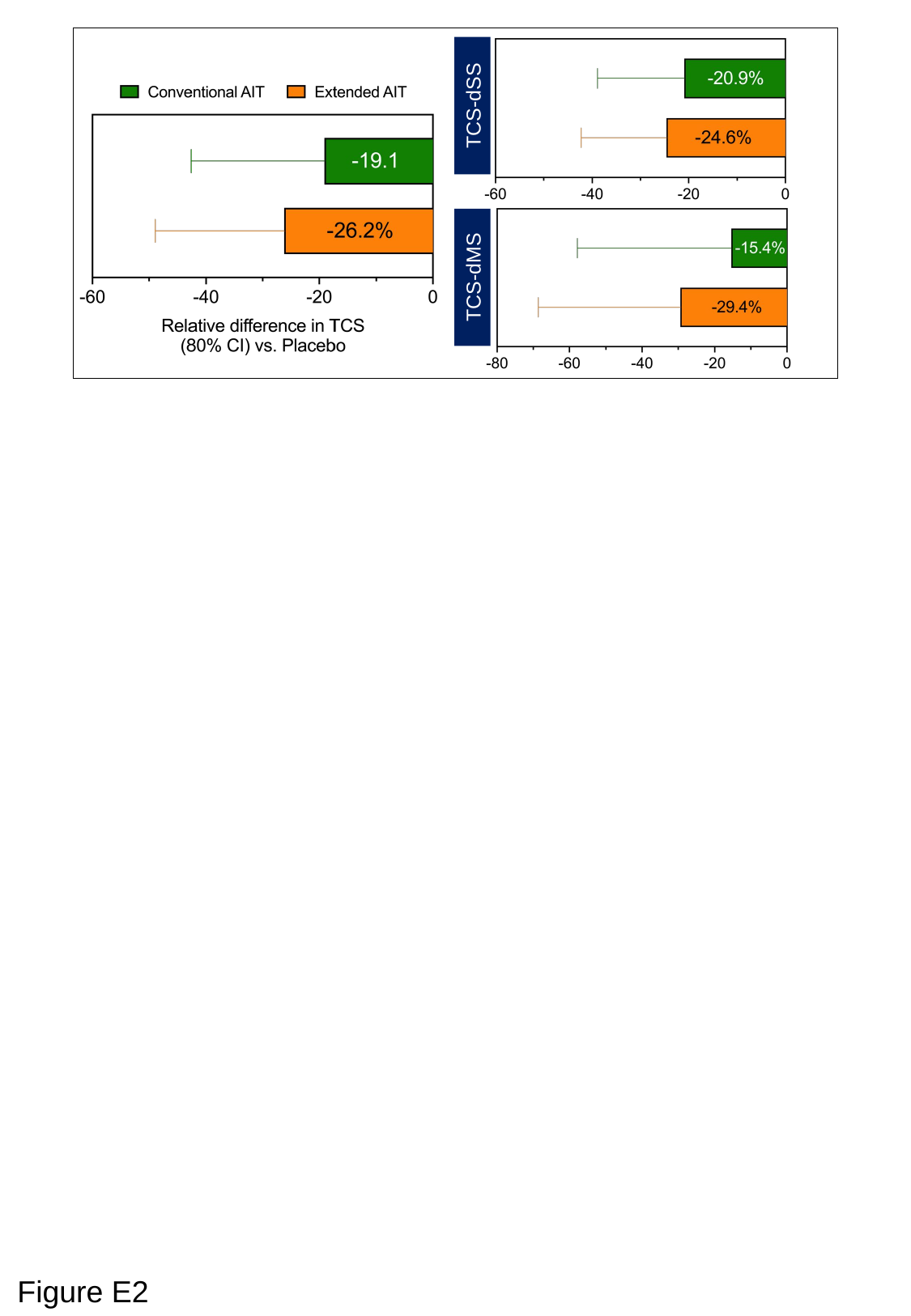

TCS-dSS
TCS-dMS
Figure E2

Supplement: Supplementary file 3 — Figure S2. [file ALL-81-232-s002.pptx]
