# Supplementary material for: Modulation of Cellular, Molecular, and Humoral Responses by PQ Grass 27,600 SU for the Treatment of Seasonal Allergic Rhinitis: A Randomised Double Blind Placebo Control Exploratory Field Study
Source: Allergy. 2025 Jul 8;81(1):232–47. doi: 10.1111/all.16640 (PMC12773655; doi:10.1111/all.16640)
Supplement: Supplementary file 4 — Figure S3. [file ALL-81-232-s003.pptx]

## Slide 1
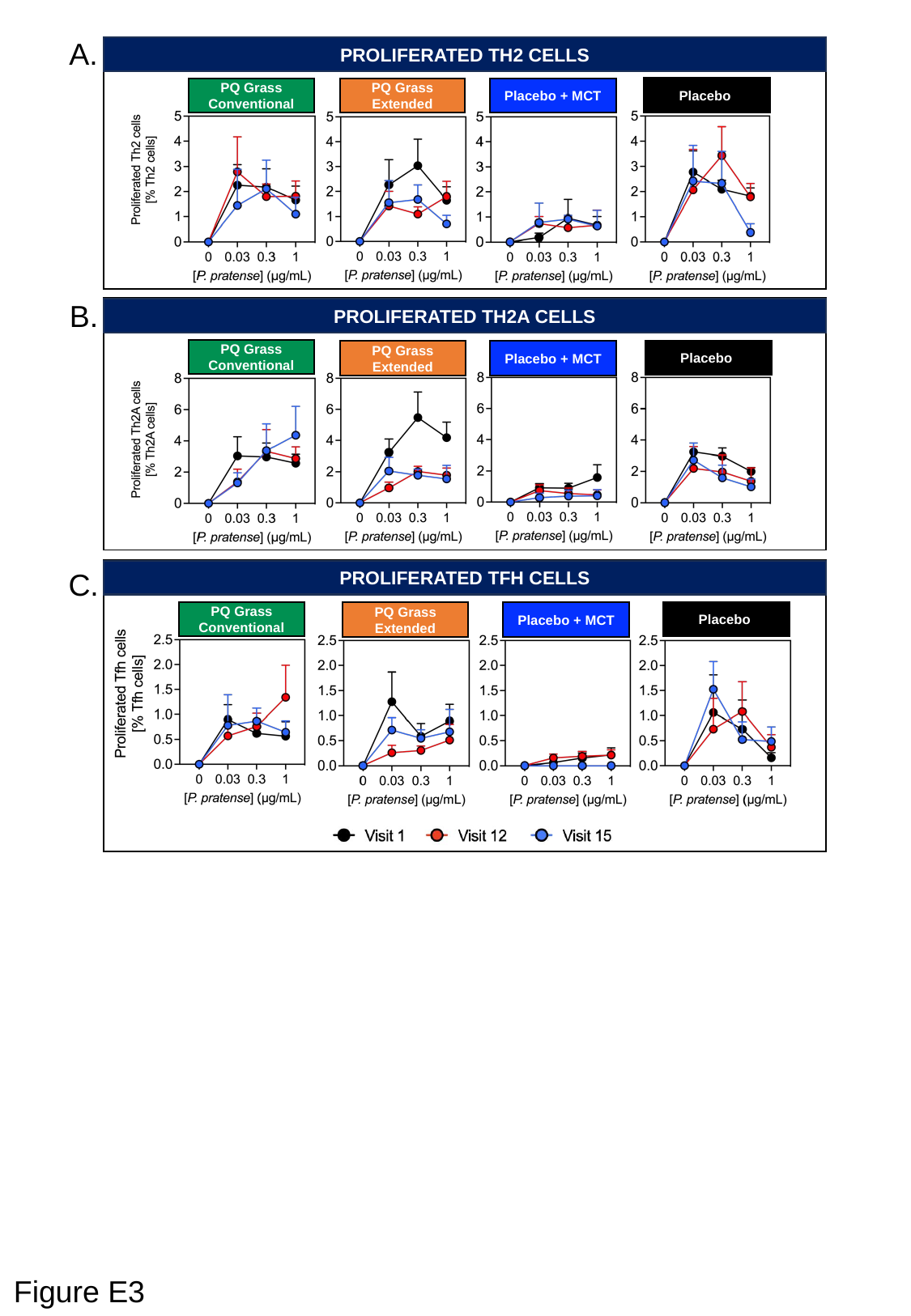

A.
PROLIFERATED TH2 CELLS
Placebo
Placebo + MCT
PQ Grass Conventional
PQ Grass Extended
B.
PROLIFERATED TH2A CELLS
PQ Grass Conventional
Placebo
Placebo + MCT
PQ Grass Extended
C.
PROLIFERATED TFH CELLS
Placebo
Placebo + MCT
PQ Grass Conventional
PQ Grass Extended
Figure E3
